# Versatility of Evaporation-Induced Self-Assembly (EISA) Method for Preparation of Mesoporous TiO_2_ for Energy and Environmental Applications

**DOI:** 10.3390/ma7042697

**Published:** 2014-03-31

**Authors:** Luther Mahoney, Ranjit T. Koodali

**Affiliations:** Department of Chemistry, University of South Dakota, Vermillion, SD 57069, USA; E-Mail: luther.mahoney@usd.edu

**Keywords:** mesoporous titanium dioxide, evaporation-induced self-assembly, non-ionic surfactants, cationic surfactants, dye sensitized solar cells, photocatalytic degradation, water splitting, batteries

## Abstract

Evaporation-Induced Self-Assembly (EISA) method for the preparation of mesoporous titanium dioxide materials is reviewed. The versatility of EISA method for the rapid and facile synthesis of TiO_2_ thin films and powders is highlighted. Non-ionic surfactants such as Pluronic P123, F127 and cationic surfactants such as cetyltrimethylammonium bromide have been extensively employed for the preparation of mesoporous TiO_2_. In particular, EISA method allows for fabrication of highly uniform, robust, crack-free films with controllable thickness. Eleven characterization techniques for elucidating the structure of the EISA prepared mesoporous TiO_2_ are discussed in this paper. These many characterization methods provide a holistic picture of the structure of mesoporous TiO_2_. Mesoporous titanium dioxide materials have been employed in several applications that include Dye Sensitized Solar Cells (DSSCs), photocatalytic degradation of organics and splitting of water, and batteries.

## 1. Introduction

Porous materials play an important role in society from the many versatile applications, as in catalysis, adsorbents, optics, sensors, insulation coatings, ultra-low density materials, *etc.* [1,2]. In the field of catalysis, porous materials are extensively used, and they directly impact the world’s economy by permitting reactions to occur under conditions that require less energy, such as in petroleum refining where various microporous zeolites play a prominent role in catalytic cracking reactions [3,4]. One of the major drawbacks of microporous materials, such as zeolites, is that their small pore openings prevent their use in demanding petroleum-refining applications [3,4]. Bulky organic molecules with molecular dimensions larger than the pore openings of zeolites cannot enter microporous structures and partake in organic transformations pivotal in pharmaceutical industry.

The three classes of porous materials, according to the International Union of Pure and Applied Chemistry (IUPAC) definition, include the following: microporous (<2 nm); mesoporous (2–50 nm); and macroporous (>50 nm) materials [5]. Microporous materials have relatively small pore sizes, such as with the zeolite family. However, mesoporous materials have the potential to remove these barriers due to their large pore diameters (2–50 nm), large pore volumes, and ordered pore channel arrangements. Moreover, the high surface and large pore volume provide an avenue to disperse active sites. The final class, macroporous materials have very large pore diameters but usually low surface areas and are employed in photonic applications, such as waveguides.

This review will focus on the synthesis, characterization, and applications of titanium dioxide mesoporous materials made by the Evaporation-Induced Self-Assembly (EISA) method. Other types of approaches for preparation of different mesoporous oxide materials including EISA are available in the following reviews [6–9]. This review is broken down into four main sections and starts with a brief history of the development of mesoporous materials, and then moves onto a description of the EISA method for the preparation of mesoporous TiO_2_. Then, characterization of TiO_2_ by a variety of techniques is provided so that researchers have a broad understanding of their physico-chemical properties and may choose to characterize the materials depending on the availability and necessity. In the final section, the applications in the area of energy and environmental technologies are discussed.

### 1.1. Development of Mesoporous Materials

**Figure 1.**
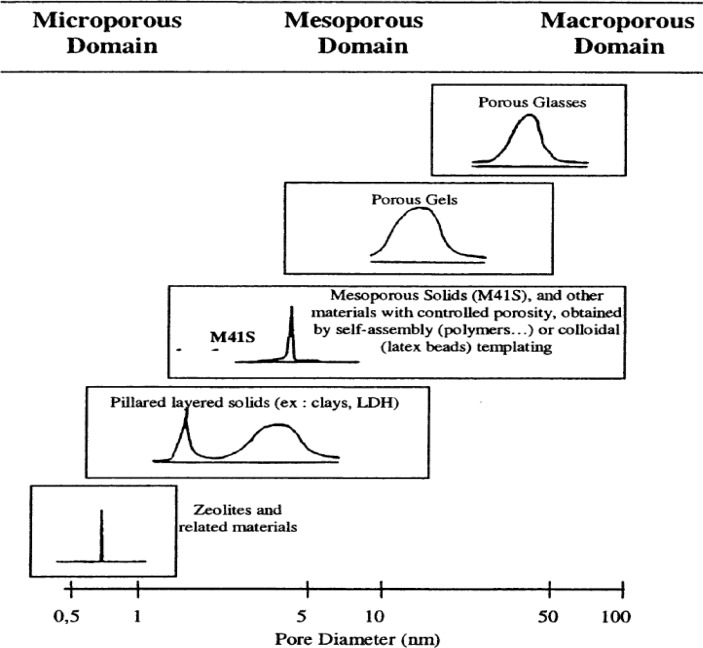
Graphical description of the various regions that porous solids occupy with regards to their pore sizes. Reprinted with permission from [1]. Copyright 2002 American Chemical Society.

The development of mesoporous materials occurred with the invention of M41S series of materials by Mobil Company researchers in 1992 [10,11]. The hydrothermal temperatures for producing these mesoporous silica materials was relatively mild with typical temperatures near 100 °C [1]. Before the early 1990s, there has been much desire to develop porous materials with larger pore diameters compared to the zeolite family of molecular sieves, so that diffusional limitations that restrict the use of large bulky organic substrates could be overcome [1–4]. The main research to expand the pore sizes of zeolites involved the use of small organic additives such as tetramethylammonium hydroxide, tetraethylammonium hydroxide, *etc.* [1]. The use of long chain surfactant molecules such as cetyltrimethylammonium bromide (CTAB), a cationic surfactant opened the way for producing highly ordered silica porous structures with pores transcending into the mesopore regime [1–4]. The M41S family of molecular sieves have a relatively narrow pore size distribution similar to zeolites, but the M41S materials have increased pore diameters compared to zeolites as shown in Figure 1. Figure 1 also shows the pore diameters of various types of porous materials. In contrast, clays and layered double hydroxides (LDH) have a combination of semi-narrow and broad peaks with slightly greater pore sizes than zeolites. Porous gels and glasses have both large and broad pore sizes in the meso- and macroporous regions. These are typically prepared without any structure directing agents or surfactant templates. The three original M41S mesoporous silica based materials include MCM-41, MCM-48, and MCM-50 [10,11]. The M41S mesoporous materials have large surface area values of greater than 500 m^2^/g, pore volumes higher than 0.5 cm^3^/g, and tunable pore sizes in the 2–10 nm range. Since the initial development of the M41S family of molecular sieves, other silica based mesoporous materials denoted as hexagonal mesoporous silica (HMS) [12], Michigan State University (MSU) series of materials [13,14], Santa Barbara Acidic (SBA) [15], FDU [16], and Mesostructured Cellular Foam (MCF) family of molecular sieves have been prepared [17,18].

### 1.2. EISA Process

The groups of Brinker and Ozin first reported the EISA process [19,20]. The EISA process was originally designed for the preparation of mesostructured silica thin films and several papers including reviews are available [1,6,21–29]. In recent years, a number of mesostructured materials have been prepared by combining sol-gel chemistry with surfactants. The sol-gel process is a facile method for the preparation of solid metal oxides and hydroxides. In this method, solutes, (precursors) are dispersed in a solvent (usually alcohol) at relatively low temperatures (<100 °C). In the sol-gel process, independent colloidal particles are first formed and often these particles are well dispersed in the solvent, and a colloidal solution or suspension called a sol is obtained. With time, the colloidal particles aggregate to form a three-dimensional open network called a gel. The two important reactions in the sol-gel process are hydrolysis and condensation, and they lead to the formation of M–OH–M or M–O–M bridges.

Amphiliphilic surfactant molecules self-assemble into various structures. The self-assembly process involves the natural and spontaneous ability of surfactants to organize themselves into different shapes and involve interactions such as Van der Waals and electrostatic forces, hydrogen bonding and π–π interactions. At a certain concentration, called the critical micelle concentration (cmc), the surfactants assemble into spherical micelles and at higher concentrations, cylindrical micelles are formed. Two mechanisms have been proposed for the formation of periodic mesoporous materials using ionic surfactants. The Liquid Crystal Templating (LCT) mechanism was first proposed by Kresge *et al*. [10] in which a stable surfactant mesophase (perhaps mediated by the inorganic anions) is first formed. The inorganic phase then undergoes condensation around the surfactant phase. In the Cooperative Self-Assembly (CSA) mechanism, the inorganic species and the surfactant molecules react first. The cooperative nucleation and aggregation leads to the formation of an organic-inorganic hybrid structure. The elimination of the surfactant molecules leads to the formation of a mesoporous material. The synthesis of periodic mesoporous materials involving such hybrid structures is perhaps a combination of these two mechanisms.

In the formation of mesoporous thin film materials, by the EISA process, a homogeneous solution consisting of surfactant, soluble metal alkoxide or metal salt, alcohol (typically ethanol), water, and often acid (usually HCl) are mixed thoroughly. The initial concentration of the surfactant in this solution is much lower than cmc. The solution is cast on a substrate by spray, spin, or dip coating and evaporation of the volatile components (especially alcohol, HCl, and water) takes place at the interface between air and the film. During the initial stages (10–30 s), because of preferential evaporation of the alcohol, the concentrations of the metal oxide oligomers and the non-volatile surfactant increases. This increase in the surfactant concentration drives the self-assembly of the organic-inorganic hybrid into a Liquid Crystal (LC) phase. With time, as the concentration of surfactant equals that of cmc, micelles are formed and an organized LC mesophase in which the inorganic network is not fully condensed called a Modulable or Tunable Steady State (MSS/TSS) is formed. In this stage, water and solvent molecules in the films are in equilibrium with the environment and they can enter or depart. The duration of MSS/TSS depends on the Relative Humidity (RH) conditions and can last anywhere from a few seconds to minutes for silica systems and for up to several hours for titania. In the final step, the template is eliminated to impart porosity and fully condense the inorganic network. Typically, calcination in the temperature range of 400 to 550 °C is performed to remove the surfactants. Mild temperature treatments such as solvent extraction, or a combination of UV-ozone have also been attempted to eliminate the surfactant molecules. The final mesostructure is dependent on three important factors, the ratio of the surfactant to the metal oxide precursor, the nature of the precursor, and RH value.

**Figure 2.**
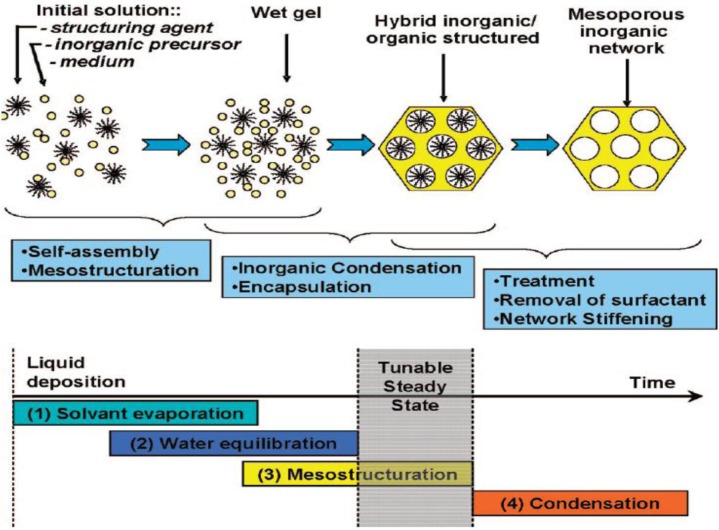
The scheme shows depictions of the various steps involved in the Evaporation-Induced Self-Assembly (EISA) process. Reprinted with permission from [26]. Copyright 2008 American Chemical Society.

The transformation of the initial solution to the final inorganic mesoporous material can be better understood from Figure 2. Figure 2 indicates the various stages involved in the formation of thin films using the EISA process. The preparation of a highly ordered mesostructured film involves four stages: (1) the evaporation of solvent that triggers the self-assembly process; (2) equilibration of water and solvent from the film with the environment; (3) the formation of an organic-inorganic mesostructure; and (4) condensation of the inorganic precursor to form a mesoporous network.

The EISA process is a simple, efficient, and rapid method for the preparation of highly uniform and robust thin films. Thin films are used in diverse applications such as separation, optics, catalysis, low dielectric layers, photovoltaics, and sensors to name a few. A big advantage of EISA process when combined with dip-coating is the flexibility afforded in controlling the final mesostructure of the thin film by careful control of processing parameters such as temperature, time, RH, vapor pressure and chemical parameters such as composition and pH.

The EISA process has also been effective in producing ordered metal oxide thin films or powders, such as TiO_2_, the focus of this review. In addition, although other metal oxides such as ZrO_2_, Al_2_O_3_, Nb_2_O_5_, Ta_2_O_5_, WO_3_, HfO_2_, and SnO_2_ *etc.* have been produced by Stucky and co-workers [30] using non-ionic surfactants, there have been issues with reproducibility in the synthesis of several of these oxides.

## 2. Synthesis

In this section, we will discuss the factors that affect the formation of periodic TiO_2_ mesoporous materials using the EISA method in greater depth. A brief discussion of the surfactants will also be provided. We will also discuss the formation of thin films and powders separately in this section. This will provide the reader with valuable information regarding the difficulties in obtaining highly ordered and periodic titania based films and powders.

### 2.1. Non-Ionic Surfactants

The various categories of non-ionic surfactants can be broken down into diblock and triblock co-polymer surfactants [1]. Diblock co-polymers are made of two repeating units: ABAB [1,31–34]. In contrast, triblock co-polymer is comprised of repeating ABA units. These co-polymers may have different combinations of hydrophilic and hydrophobic components so that their interactions with the metal species can be controlled. Examples of hydrophilic blocks include polyethylene oxide (PEO) and polyacrylic acid (PAA). Hydrophobic units involve one or several of the blocks: polystyrene (PS), polypropylene (PPO), polyisoprene (PI), or polyvinylpyrrolidone (PVP). With this knowledge, we will survey the many combinations of non-ionic surfactants employed to produce mesoporous TiO_2_ materials. Examples of hydrophilic non-ionic co-polymer surfactants with hydroxyl group capped polar groups of the formula C*_n_*H_2_*_n_*_+1_[EO]*_m_*OH include the following: Tergitol, Triton, Igepal, and Brij. These surfactants contain several hydroxyl groups, which make them hydrophilic. The majority of mesoporous TiO_2_ synthesized with non-ionic surfactants employ a pluronic co-polymer that have the formula of [PEO]*_n_*[PPO]*_m_*[PEO]*_n_*. Examples of common pluronic co-polymers include the following: P123, and F127; other non-ionic surfactants with different types of hydrophobic groups include, such as polyisobutylene [1,34]. The pluronic P123 has the formula, (PEO)_20_(PPO)_70_(PEO)_20_ polymer while F127 has the formula (PEO)_106_(PPO)_70_(PEO)_106_ [31]. From reviewing the different publications employing P123 or similar non-ionic surfactants, one notices that the periodicity (ordering) of the pores are relatively low [35–40]. However, in recent years, improvements in pore ordering have been obtained using these non-ionic surfactants [41–45]. Furthermore, the use of non-ionic surfactants such as F127 in general produced well-ordered TiO_2_ structures [46–53]. Moreover, studies employing custom tailored non-ionic surfactants, such as poly(ethylene-co-butylene)-*b*-poly(ethylene oxide), also known as KLE produced well-ordered pore structures of TiO_2_ [54–56]. These custom made non-ionic surfactants have more hydrophobic character compared to the pluronic P123 surfactant. This increases the stability of these micelles in less polar solvents such as ethanol. An added advantage of this surfactant is that they have very low polydispersity and thus form spherical micelles exclusively [57]. Thus, it is tempting to conclude that the degree of hydrophobicity directly influences the quality of the mesostructure formed. However, one must exercise caution in coming to this conclusion because the impact of other variables must also to be considered.

### 2.2. Cationic Surfactants

The main types of cationic surfactants include alkyltrimethyl quaternary ammonium, gemini, bolaform, tri-head group, and tetra-head group rigid bolaform [2]. The alkyltrimethyl quaternary ammonium cationic surfactants are comprised of a long alkyl chains ranging from 8 to 22 methylene units (–CH_2_–) connected to a terminal –CH_3_ group and typically contains three methyl groups linked to the N atom. One of these methyl groups can be substituted with long chain alkyl groups containing methyl or aromatic groups at the end. The next most common cationic surfactants are the gemini series. These cationic surfactants contain methylene linkages ranging from 2 to 6 and connecting the two alkyl ammonium groups. Variations in the chain length of the alkyl groups leads to a wide range of gemini surfactants. Bolaform cationic surfactant class is similar to the gemini surfactant except that the linkages between the two alkyl ammonium groups are aromatic in nature; this causes this surfactant to have additional rigidity. Finally, the alkyltrimethyl quaternary ammonium surfactant comprised of 16 carbons known as CTAB is the most common cationic surfactant used in producing mesoporous TiO_2_, and this may attributed to the relatively low cost and availability. CTAB has a few advantages compared to the non-ionic surfactant, Pluronic P123 and F127. The cmc value of CTAB is greater than that of P123 or F127, which means that CTAB solutions can be used at room temperature with relatively large amounts dissolving in an aqueous phase [58–60]. In addition, the critical micelle concentration (cmc) is lower for CTAB compared to other non-ionic surfactants, such as P123 and F127. The cloud point is a major issue with non-ionic surfactants and hence careful adjustment of the temperature is necessary to dissolve the non-ionic surfactant, but one has to be careful to not raise the temperature too greatly above ambient conditions, thereby causing the surfactant to precipitate out of solution [2]. This dissolution challenge with non-ionic surfactants is not an issue for cationic surfactants, such as CTAB. Moreover, CTAB can be used in both acidic and basic synthesis conditions. However, the major challenge for use of cationic surfactants, such as CTAB, is the toxicity and relatively higher cost compared to non-ionic surfactants. In the next section, we will discuss the synthesis of mesoporous titania films.

### 2.3. Synthesis of TiO_2_ Mesoporous Thin Films via EISA Process

The EISA method for preparation of mesoporous titania films is more challenging compared to the formation of silica. This can be traced to the high electrophilicity of Ti^4+^ ion compared to Si^4+^. Therefore, steps must be taken to prevent nucleophiles, such as water, from easily attacking the Ti^4+^ ion to prevent uncontrolled hydrolysis and condensation that lead to the formation of poorly structured and dense inorganic networks. In addition, removal of the surfactant template by calcination poses challenges because the oxides tend to crystallize, which tends to collapse the mesopores.

**Figure 3.**
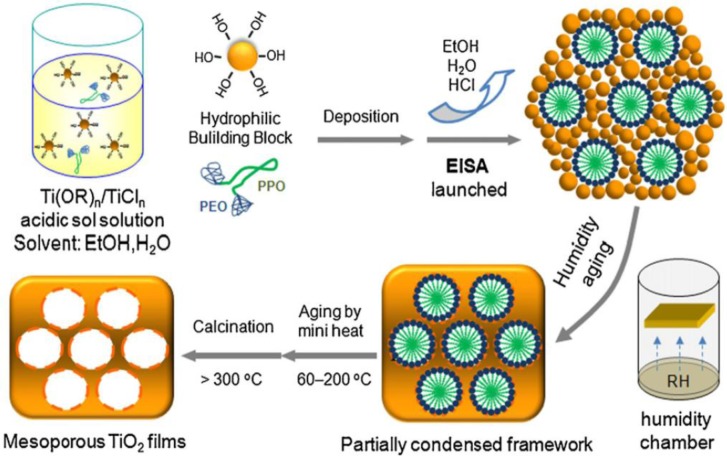
Schematic representation of the formation of mesoporous titania thin film. Reprinted with permission from [28]. Copyright 2011 Elsevier.

Figure 3 illustrates the EISA process for the synthesis of mesoporous titania films. The initial solution consists of a mixture of titania precursor (Ti(OR)_4_ and/or TiCl_4_), solvent (alcohol), acid, and a surfactant, such as block copolymer. The deposition of the sol leads to the formation of a titania-surfactant hybrid phase. The titania phase containing terminal hydroxyl groups act as Nano Building Blocks (NBB) and do not undergo hydrolysis easily. They interact with the hydrophilic portion of the block copolymer through H-bonding interactions. In the next step, the films are aged during which condensation of the titania network occurs, and this leads to the formation of a highly organized mesostructure. In the final step, the template is removed, usually by calcination to yield mesoporous films.

Sanchez and co-workers reported the preparation of a uniform and 2D-hexagonal mesostructured titania thin films using TiCl_4_, Pluronic F127 or Brij 58 as the surfactant and ethanol as the solvent. The thin films retained their periodicity even after calcination at 350 °C [61]. Key factors that helped retain the periodicity was the use of moderate amounts of water ([H_2_O]/Ti < 10), the *in situ* generation of large quantities of HCl that allowed the hydrolysis-condensation reactions to proceed in a controlled manner and ammonia treatment prior to calcination in air. Chemical analysis and Rutherford Back Scattering (RBS) studies indicate the formation of titanium-choloro-alkoxy species (TiCl_4−_*_x_*(OC_2_H_5_)*_x_*, *x*~2) that act as building blocks of the inorganic walls. In a follow up study, the chemical and processing parameters were optimized for the preparation of highly oriented 2D hexagonal (*p*6*m*), or 3D cubic (*Im*3*m*) titania mesostructures [62].

Sanchez and co-workers studied six variables that affect the quality of TiO_2_ films, and they found that the RH value has a dramatic result on the structure of the final material [62]. The six variables examined included the following: (1) type of surfactant; (2) the ratio of surfactant to titania precursor; (3) volume of water added into the reaction mixture; (4) amount of moisture or water vapor (RH) in the environment; (5) the solution pH (that could be adjusted by controlling the ratio of the titania precursors, *i.e*., TiCl_4_/Ti(OEt)_4_; and (6) the reaction temperature during both the synthesis and aging processes.

**Figure 4.**
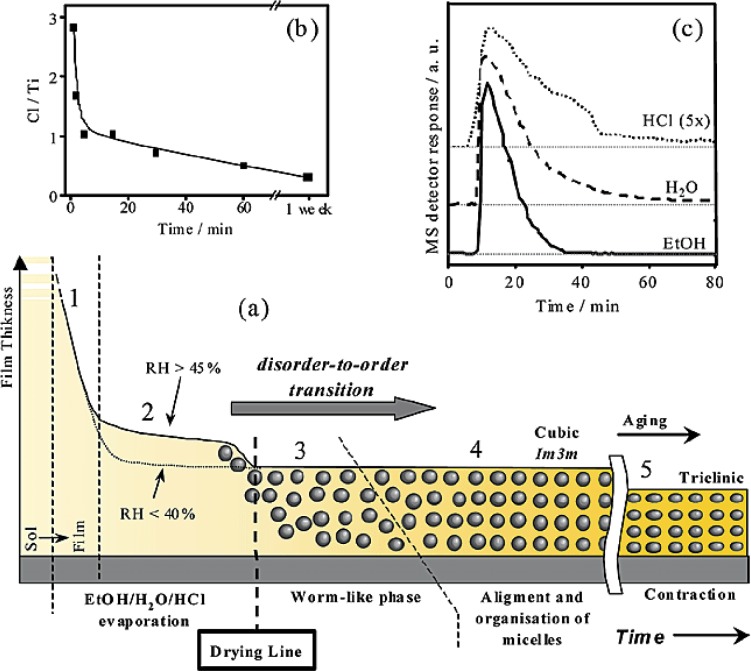
(**a**) Depiction of the progress from disordered to highly ordered structure using non-ionic Brij 58 surfactant; (**b**) EDX analysis of the Cl/Ti ratio as a function of time; and (**c**) evolution of volatile species from mass spectrometry. Reprinted with permission from [62]. Copyright 2003 American Chemical Society.

Figure 4 shows the various steps in forming ordered mesoporous TiO_2_ structure beginning with the surfactant solution. Figure 4 shows five regions. The first region involves the evaporation of ethanol vapors from the reaction mixture and the second region involves release of water-HCl mixture into vapor phase. During these two regions, there is a decrease in the thickness of the film as a consequence of evaporation of the volatile components. The third can be detected only when RH ≥ 45%. Small Angle X-ray Scattering (SAXS) indicates a broad diffusion ring in this region indicating the presence of a disordered or worm-like phase. At this time, micelles are formed but are randomly oriented. In the fourth region, organization and alignment of the micelles occurs and SAXS studies indicate well-defined diffraction peaks. The fifth and last region shows the removal of remnant water vapor and HCl leading to further condensation of the mesoporous TiO_2_ structure.

SAXS experiments indicate the importance of RH in the transformation of the disordered state to an ordered one. When RH was less than 20%, a worm-whole like phase was obtained whereas titania mesostructures with increasing periodicity and organization was obtained as the RH value increased. Cubic titania films were obtained at high RH values; however, hexagonal films were obtained at lower RH values. The presence of water creates sufficient “fluidity” or flexibility to permit the organization of the template to an ordered state. The role of water (in solution and vapor) is to enhance the hydrolysis rate of the titania precursor and make it hydrophilic so as to foster their interaction with the hydrophilic portion of the block copolymer. Also, an increase in water will favor condensation of the titania network.

Pan and Lee discovered that an optimal water concentration is effective in obtaining hexagonal and cubic titania mesostructured films [49]. When the molar ratio of the titanium isopropoxide to water was maintained at 20, rapid hydrolysis of the titanium precursor destroyed the periodicity of the mesostructure. Interestingly, they observed that the mesophase could be controlled by the speed of the spin-coating process. At low speeds (near 600 rpm), cubic phases were obtained. At intermediate speeds (1000 rpm), a mixture of cubic and hexagonal phases were obtained and at speeds of 2000 rpm, hexagonal phase was obtained. The variation in the speed of spin-coating leads to differences in the evaporation rate of ethanol. At low speeds, the evaporation of ethanol is relatively slow and hence an ethanol-rich environment exists; under such conditions, cubic phase is formed, whereas an ethanol-deficient environment is formed when the spin-coating speed is increased. This leads to the formation of a hexagonal phase. Lu and co-workers found that the crystallite size of anatase, defect density, and mesophase could be controlled by the amount of water [63].

The choice of solvent used is also critical. The reason that an alcohol is employed is that it is relatively a weaker nucleophile compared with water and therefore, one can control the hydrolysis reaction better when a water-alcohol mixture is used instead of water only [22,26,28]. Also, alcohols are highly volatile and their large wetting ability to hydrophilic substrates produces consistent and uniform films, and this is the main reason why ethanol is usually employed in many EISA syntheses. A thermally stable and 2D hexagonal mesoporous nanocrystalline anatase with robust anatase pore walls was prepared by using 1-butanol as the solvent [35]. The higher hydrophobicity of 1-butanol in comparison to ethanol enhances the microphase separation between the surfactant P123 and the aqueous phase. Thus, the addition of 1-butanol favors the formation of micelles of the P123 template. An added advantage of 1-butanol is the increase in the mesopore diameter due to an increase in the volume of the hydrophobic core. 1-butanol can effectively penetrate the poly(propylene oxide) core.

Chelating ligands like acetylacetonate (acac) have been used to modulate the fast hydrolysis rate of titanium alkoxides for the preparation of TiO_2_ thin films. The chelating ligand reduces the positive character on the Ti cation center, thereby reducing the effects of a nucleophilic attack from water. Lu and co-workers [63] used acac to produce mesoporous TiO_2_ with non-ionic surfactant, and they obtained hexagonal ordered pores by calcination at 300 °C for 4 h. Similarly, Dai and co-workers [64] reacted titanium butoxide (TBT) with acac to form mesoporous TiO_2_ thin films and lattice fringes were observed in their transmission electron microscopic images, which imply crystallinity of the titania films. However, the pore ordering was dramatically reduced. Sanchez and co-workers [65] found that titanium butoxide (TBT), titanium isopropoxide (TPT), and titanium ethoxide (TET) had different reactivities toward nucleophiles, such as water. These researchers noted lower reactivity of TBT when compared with TPT, and attributed this to steric effect due to the presence of bulkier butoxy groups, which restricts the attack of water to the electrophilic Ti^4+^ center. A large-pore (14 nm) mesoporous nanocrystalline titania thin film was prepared using TBT and P123 by Fu and co-workers [36]. Furthermore, the pore sizes could be tuned by careful adjustment of the TBT/P123 ratio. 1-butanol that is produced *in situ*, acts as a co-surfactant and swelling agent and an increase in TBT leads to the formation of larger amounts of 1-butanol and hence an increase in the pore size is obtained. Titanium alkoxides have been the popular choice as the titania precursor, but TiCl_4_ has also been employed as a precursor for the formation of well-defined mesophase [61,66,67]. In the next section, we will discuss the film processing methods.

#### Film Processing

**Figure 5.**
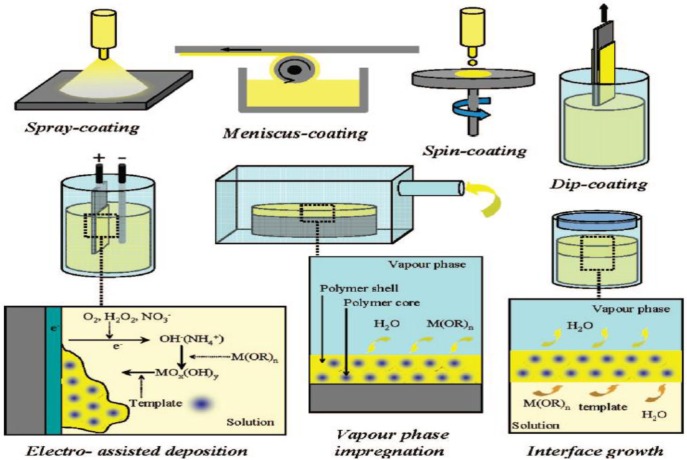
The scheme shows the many avenues for producing ordered mesoporous TiO_2_ films. Reprinted with permission from [26]. Copyright 2008 American Chemical Society.

The EISA method is a convenient and facile method for preparation of mesoporous thin films. The depositing of the sol onto substrate can be completed using several different methods, as illustrated in Figure 5 [26]. The four depositing methods including spray-coating, meniscus-coating, spin-coating, and dip-coating are readily adaptable to the EISA synthetic method and are shown at the top in Figure 5.

The other film forming methods, such as electro-assisted deposition, vapor phase impregnation, and interface growth, are not widely used in the EISA method; rather, they are film depositing methods, which have reduced control of synthesis variables for formation of mesoporous structures. Therefore, the film forming methods that will be described are the ones related to the EISA synthesis. The spray-coating deposition method employs the aerosol method to form films on a substrate. The meniscus-coating film forming method begins by placing a drop in solution and permitting it to spread over the liquid phase, thereby forming a film after adequate aging period. The spin-coating film depositing method starts by placing a drop onto a substrate followed with centrifugal force causing the initial drop to expand over the substrate depending on the number of revolutions-per-minute employed. The final EISA method commonly employed is the dip-coating of substrate in which a sol containing the precursors are placed in a vessel and a substrate is withdrawn slowly. These four methods discussed are adaptable to the EISA method, and they have been shown to produce high quality TiO_2_ films.

The non-ionic surfactant interfacial chemistry involves hydrogen bonding, van der Waals forces and weak coordination bonds between the titanium ions and the hydrophilic poly(ethylene oxide) moieties. Successful preparation of well-defined titania thin film mesophases have been obtained with P123, F127, Brij 58, and KLE as structure directing agents [35,49,62,66–80]. Ionic surfactants such as CTAB have been extensively used to synthesize mesoporous titania powders, the topic of discussion in the next section. Also, the EISA synthesis for the preparation of mesoporous titanium dioxide does not typically involve the use of anionic surfactants because they bind very strongly to the highly electrophilic titanium precursor.

### 2.4. Synthesis of TiO_2_ Mesoporous Powders

**Figure 6.**
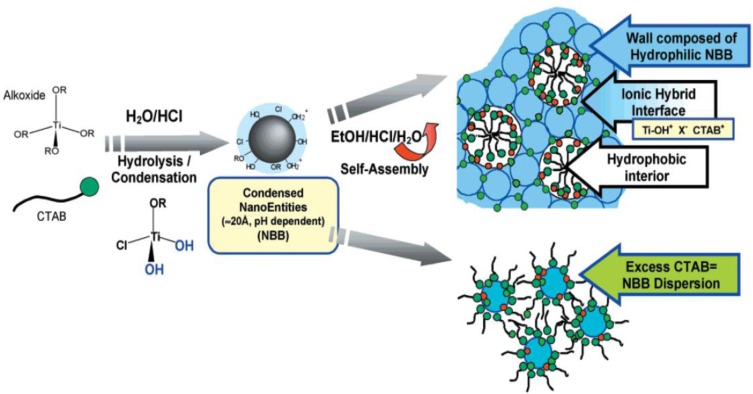
Figure depicts the synthesis of mesoporous TiO_2_ using cationic cetyltrimethylammonium bromide (CTAB) surfactant. Reprinted with permission from [83]. Copyright 2002 American Chemical Society.

Stucky and co-workers [30,81] reported the synthesis of large-pore mesoporous metal oxide powders and films, using P123 surfactant. Inorganic metal chloride salts were used as precursors in their studies. A non-hydrolytic route that involves carbon-oxygen bond cleavage was proposed to delay the crystallization of titania, and this appeared to be critical in the preparation of the mesostructures in a controlled manner. The role of water was probed in detail by Sanchez and co-workers [82] using TBT, TET, or TPT as precursors. At low water contents, the rate of condensation is very low and does not occur before the formation of the mesostructured hybrid phase. On the other hand, in the presence of large amounts of water, condensation reaction leads to the formation of oxo clusters prior to the formation of hybrid phase. However, if excess water was added, gels that lacked periodicity were formed. Mesoporous titania using CTAB and TET were prepared using the EISA approach. The hybrid “titaniatropic” phase consists of NBB, which self-assemble around the micelles. This assembly can be considered to have interactions of the type, Ti–OH^+^…–X^−^…CTAB^+^, where X represents bromide anion from CTAB, or chloride ions from HCl used in the synthesis. Figure 6 shows the idealized synthesis of mesoporous TiO_2_ using cationic CTAB surfactant [83]. As depicted in Figure 6 when too much CTAB is added, a dispersion of NBB is formed.

Yan and co-workers [46] discovered that by changing the solvent and co-solvent different phases of titania could be obtained. Using TiCl_4_, P123 or F127 as the structure-directing agent, and by varying the solvent from methanol, to ethanol, 1-butanol and 1-octanol, anatase, a mixture of anatase and rutile, and pure rutile was obtained respectively. The differences were explained on the leaving ability of chlorine atoms. When the carbon chain increase, more chlorine tends to stay in the choloro-alkoxide [TiCl_4−_*_x_*(OR)*_x_*] moieties due to an increase in the steric hindrance. Low acidity tends to form anatase whereas high acidity forms rutile phases. A mixture of TiCl_4_ and TBT along with P123 was used to form a well-ordered mesostructure of TiO_2_ [84]. Interestingly, pore walls with mixed phases of anatase and rutile were obtained in this study. By adjusting the relative amount of the starting precursor, TiCl_4_, different amounts of anatase and rutile phases were obtained. Porous titanium dioxide with worm-hole like structure was obtained using TET and CTAB [85]. A highly ordered mesoporous titania was prepared by using TiCl_4_ and P123 [51]. Transmission electron microscopic images however indicate the presence of both ordered and worm-hole phases in these materials. In addition, in the absence of low angle powder X-ray diffractometry (XRD) studies, it is not clear if these materials possess periodicity. Furthermore, the wide-angle XRD studies show only a weak and broad peak near 2θ = 25° indicating poor crystallinity. A series of 2D-hexagonal titania mesoporous materials were prepared by varying the ratio of P123 to TBT. The mesoporous material prepared at a P123/TBT mole ratio of 0.2 possessed a surface area of 244 m^2^/g [86]. Citric acid was added to a mixture of TPT and F127 to form mesoporous titania [52]. Citric acid functionalizes the surface of the hydrophilic titania nanoparticles and enhances the binding of these particles to the ethylene oxide units of F127. A thermally stable mesoporous anatase was prepared by adding ethylenediamine (en) after the titania-P123 mesostructure was formed. The en molecules bind to the surface of titania nanoparticles and prevent collapse of pores, also preventing the transformation of anatase to rutile despite the use of calcination temperatures as high as 700 °C [43].

**Figure 7.**
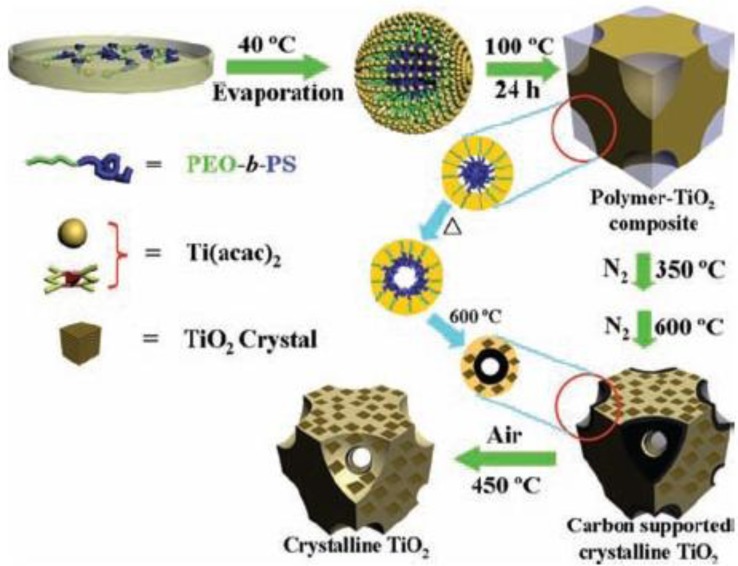
Scheme showing the positive effects of multistep heat treatment in forming large-pore mesoporous TiO_2_ materials. Reprinted with permission from [87]. Copyright 2011 John Wiley and Sons.

A ligand assisted method using PEO-*b*-PS (PS = polystyrene) and TPT was developed by Zhao and co-workers [87]. Pore sizes as large as 16 nm was obtained using this strategy as shown in Figure 7 [87].

A small, non-ionic fluorinated surfactant [C_8_F_17_C_2_H_4_-(OC_2_H_4_)_9_OH] was used for the preparation of hexagonal mesostructured titania [88]. The resulting material had a large surface area of 590 m^2^/g. A mixture of acids, HCl and H_2_SO_4_, was used by Zhou and co-workers to prepare mesoporous anatase [41]. In this method, the surfactant was carbonized by sulfuric acid to form amorphous carbon layer inside the mesochannels by heating in N_2_. This helps in preventing the collapse of the pores on further calcination to remove the carbon. The key was using a multi-step thermal treatment to remove the non-ionic surfactant.

### 2.5. Removal of Surfactants

A key step in the preparation of periodic mesoporous material is the removal of the surfactant by thermal treatment methods. Non-ionic co-polymer surfactants are usually removed by calcination at temperatures in the range of 300–350 °C. Such temperatures are preferred because excessive sintering of the titanium framework causes pore wall collapse [35,36,38,39,43,49,51,61–63,65,77,84,86,89–96]. Ozin and co-workers used P123 as the surfactant for the preparation of mesoporous TiO_2_ and calcined the material in air at either 350 °C or 400 °C and obtained reasonable hexagonal pore ordering [35]. Nevertheless, the material calcined at 400 °C had greater crystallinity, but this sample also had reduced pore ordering with a pore diameter of 6.2 nm compared to the mesoporous TiO_2_ sample calcined at 350 °C, which had a pore diameter of 7.3 nm. By using a chelating agent, such as en, Fu and co-workers [43] were able to increase the pore sizes and enhance the crystallinity of the anatase phase despite the use of higher calcination temperatures of 700 °C. It should be noted that higher calcination temperatures can cause sintering of the TiO_2_ crystallites leading to lower pore sizes, as this study showed. Pore wall collapse occurs even when non-ionic surfactants are used for the preparation of TiO_2_ mesoporous materials, especially when temperatures greater than 400 °C are used [1].

**Figure 8.**
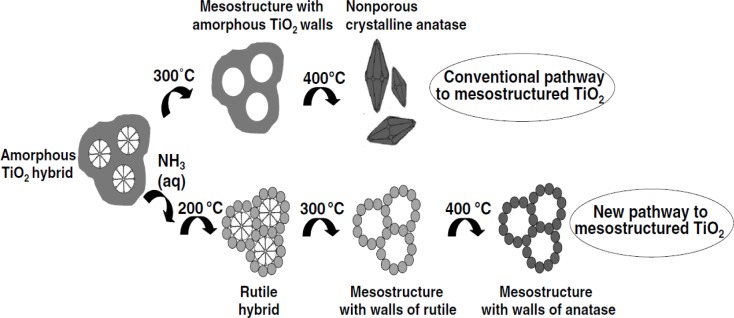
Figure shows the use of NH_4_OH to produce mesoporous TiO_2_ in EISA synthesis. Reprinted with permission from [100]. Copyright 2007 Elsevier.

The cationic surfactant CTAB requires relatively higher temperature to be removed in the calcination process and temperatures in the range of 450–550 °C are typically used [97,98]. Lower calcination temperatures have been attempted to remove the cationic CTAB surfactant [7,83,85,99,100]. However, the CTAB surfactant does not easily decompose and leave the mesoporous titanium dioxide material due to the strong Ti–OH^+^…–X^−^…CTAB^+^ electrostatic bonding interactions. Sanchez and co-workers [83] found that CTAB fragments are still present in the TiO_2_ material even after the sample has been calcined beyond 600 °C from TGA and FT-IR analysis. Thus, an appropriate calcination temperature and heating rate need to be used in the synthesis of periodic mesoporous TiO_2_ materials. A major challenge in synthesizing highly ordered TiO_2_ mesoporous structures is the formation of fairly thin walls, and their competition in forming crystalline anatase phase. Various research works have focused on ways to reduce the strain placed on the mesoporous TiO_2_ framework [1,26,28]. Fu and co-workers [43] employed pyrolysis in N_2_ flow first at 350 °C followed by calcination in air from 350 to 900 °C for 2 h. These researchers were able to retain some periodicity in the pore ordering even after calcination at 500 °C. Likewise, Herregods and co-workers [101] attempted to employ various heating periods to produce periodic ordered mesoporous TiO_2_. However, the researchers were not able to retain the periodic ordered network of pores at calcination temperatures of 440 °C. Therefore, the heat treatment step must be completed carefully to retain the periodic mesoporous TiO_2_ structure. The major methods for reducing TiO_2_ framework strain includes use of optimal heating rate, calcination temperature, and time, non-ionic surfactants and multiple heating steps. One strategy to prevent the collapse of the porous structure is to use several heating steps [1]. In addition, reduced heating rates have also been investigated in hopes of preventing pore wall collapse. Furthermore, some researchers have tried to retain the mesoporosity of titanium dioxide by simply reducing the calcination temperature or using high heating rates for short periods [55,56,93]. Reduced calcination temperature has been studied on titanium dioxide prepared using both non-ionic and ionic surfactants. Arconada and co-workers [93] used a low temperature of 350 °C for 90 min. to calcine their mesoporous TiO_2_ sample. However, reduced heating periods and lower calcination temperatures come at the expense of crystallinity. Such materials are either amorphous or have relatively low crystallinity, which is a drawback for reactions, such as photocatalytic water splitting because the recombination of the photogenerated electrons and holes are favorable when the crystallinity is low. Other researchers have used short bursts of high heating rates in hopes of obtaining high crystallinity with retention of pore ordering [55,56]. Liu and co-workers [53] were able to obtain reasonable solar hydrogen evolution production from a methanol-water mixture because the mesoporous TiO_2_ materials had relatively high crystallinity after being calcined at 500 °C for 5 h. The problem of pore wall collapse is more apparent in TiO_2_, prepared using cationic surfactants, such as CTAB because they bind strongly to the titania pore walls by Ti–OH^+^…–X^−^…CTAB^+^ type of interactions. The end result is usually the collapse of the pore structure since elevated calcination temperatures have to be employed, and the pore structure is not robust enough to withstand high calcination temperatures [85]. Also, cationic surfactants like CTAB do not form highly stable TiO_2_ porous materials from the relatively thin pore walls. The challenge with CTAB prepared mesoporous TiO_2_ materials is in removing the cationic surfactant at the lowest possible calcination temperature to prevent pore wall collapse. The aspect of low calcination conditions is illustrated in Figure 8 [100].

Calcination at 300 and 400 °C will lead to non-porous TiO_2_, due to the crystallization of amorphous titania to anatase. This invariably leads to the collapse of the pores since there is no agent to block the growth of the nuclei. Ammonia treatment, followed by calcination at 200 °C allows for the transformation of amorphous titania walls to the rutile hybrid phase first. The crystallization of the rutile phase occurs in a very controlled manner, and rapid and extensive crystallization is avoided. On heating to 300 °C, the template is removed with little or no growth in the crystallite size of rutile. A subsequent thermal treatment leads to anatase TiO_2_ with a mesoporous structure. Sanchez and co-workers [83] found that not all of the CTAB surfactant could be removed at low calcination temperatures, such as 350 °C or 400 °C. They found from DSC/TGA results that higher temperatures like 500 °C were required to remove the cationic surfactant and increase crystallinity. In addition, other researchers have used the following treatments to remove surfactant molecules from the resulting material: mild thermal treatments of 300 °C and lower [63,64,90,98,99], Ar or O_2_ plasma [102,103], and a combination of UV and ozone (O_3_) treatment [104]. Another method to remove the surfactant is by extraction of the solvent by refluxing [105] or under supercritical conditions [106]. However, the quality of some of the resulting mesoporous TiO_2_ is relatively low and these surfactant removal strategies do not always eliminate the template completely, thereby leading to pore blockage.

So far, we have discussed the mechanism involved in the EISA process and the various factoring governing the synthesis of ordered mesoporous TiO_2_ material. In the following section, we will review the various physico-chemical techniques used for characterizing the mesoporous titania material. The distinction between the characterization of mesoporous films and powders will be pointed out in this section.

## 3. Characterization

The characterization of mesoporous TiO_2_ films and powders involves using several techniques to provide a broad overview of the pore structure, level of crystallinity, and type of TiO_2_ phase. Elucidation of all aspects of the given mesoporous TiO_2_ material are best completed using many different characterization methods to get a holistic picture of the physico-chemical properties of TiO_2_. The most common characterization technique employed after the EISA synthesis of mesoporous TiO_2_ materials is powder X-Ray Diffraction (XRD) analysis. Powder XRD provides a general idea of the crystallinity, nature of the phase (anatase, rutile, brookite *etc.*), and crystallite size. In addition, by scanning the low angle regions (typically between two theta values of 1 to 5 degrees), information regarding the periodicity of the pores can be inferred [26,29]. For thin films, grazing incidence X-ray measurements are best suited to limit the incident X-rays to the surface. At incident angles, below the critical angle for reflection, the incident X-ray beam is evanescent, and thus the X-rays only sample the surface (~10 nm). Hence, the background from the substrate can be avoided. Thus, small penetration depths and an enhancement in the intensity make grazing incidence studies particularly useful for characterizing titania thin films [69]. Other techniques such as Kr and N_2_ physisorption will be noted throughout the proceeding text to delineate the textural properties (pore size, surface area, and pore volume) of the mesoporous TiO_2_ material [5,107,108]. Kr adsorption is useful for thin film materials that possess low pore volumes, whereas N_2_ gas is used for determining the textural properties of the powdered samples.

Raman spectroscopy can provide information regarding the nature of phase(s) of titania, crystallinity, and complement the powder XRD studies [109]. Small-Angle X-ray Scattering (SAXS) can be employed to elucidate the mesoscopic ordering of the mesoporous TiO_2_ films [110]. Transmission Electron Microscopy (TEM), Scanning Electron Microscopy (SEM), and Atomic Force Microscopy (AFM) studies will provide information regarding the morphology, pore sizes, particle size, and surface features of the materials respectively [26,29]. X-ray Photoelectron Spectroscopy (XPS) provides information regarding the surface composition and relative amount of surface species (for e.g., hydroxyl groups) and the presence of impurities such as residual surfactants that may be present in the pores can be easily discerned [109]. Diffuse Reflectance Spectroscopy (DRS) analysis technique provides information regarding band gap and particle sizes of titania. Fourier Transform-InfraRed (FT-IR) spectroscopy gives clues regarding the presence (if any) of surfactant remnants in the calcined mesoporous materials. Thermo-Gravimetric Analysis (TGA) and Differential Scanning Calorimetry (DSC) provide information regarding the minimum calcination temperature required to remove the surfactant molecules completely [26,29]. A combination of these several techniques will provide a comprehensive picture of the physico-chemical properties of the mesoporous material; this in turn will allow one to obtain a meaningful structure-activity correlation.

This section will start with powder XRD analysis of both low- and high-angle regions and we will then proceed to other characterization techniques sequentially. This will provide a framework for effective discussion of the application of mesoporous materials for solar energy applications, a flourishing and topical area of research.

### 3.1. Powder X-ray Diffraction (XRD)

**Figure 9.**
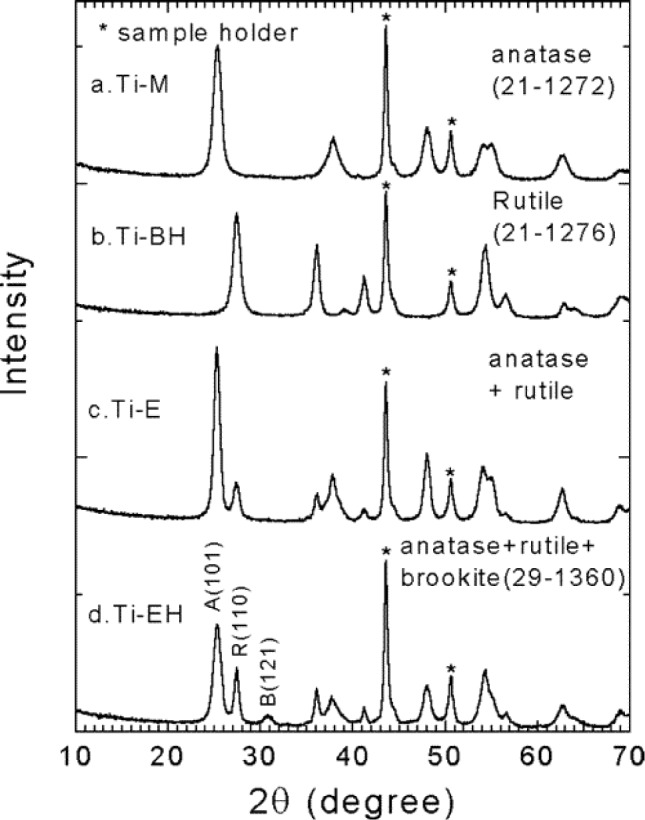
The powder XRD diffraction patterns show different phases of TiO_2_ depending on aging time and co-solvent employed. Reprinted with permission from [46]. Copyright 2003 American Chemical Society.

Powder XRD analysis is a rapid, easy, and non-destructive analysis that can provide information related to crystallinity and phase of titania [3,4]. A survey of mesoporous TiO_2_ materials made by EISA method indicates that non-ionic surfactants generally lead to well-ordered mesoporous TiO_2_ structures [35–53,95]. This can be inferred from the presence of peaks in the low angle region in the XRD scans and from the images of the pores in the TEM studies. In contrast, the mesoporous TiO_2_ materials made with cationic surfactant CTAB, lead to little to no periodic pore ordering from the weak to no peaks observed in the low-angle powder XRD plots [83,85,97,98]. Cationic surfactants, such as CTAB, have stronger ionic bonding interactions between the TiO_2_ framework and the surfactant molecules as discussed earlier. This requires elevated calcination temperatures, a process that also leads to collapse of the pores [2]. Non-ionic surfactants decompose at much lower temperatures of 300–350 °C compared with 500 °C and above for CTAB. Information about crystallinity and the phase of TiO_2_ powders can be inferred from XRD as shown in Figure 9.

In Figure 9, the aging time was varied from 3 to 7 days, and the type of alcohols were also varied [46]. In the upper plot (sample labelled, Ti–M), the co-solvent was methanol for the synthesis of anatase TiO_2_ and the aging time was 3 days at 40 °C in air. By changing the co-solvent to 1-butanol, the structure became rutile in the Ti-BH sample aged for 7 days. A combination of anatase and rutile phases were produced by simply changing to ethanol as the solvent in the Ti–E sample with an aging time of 6 days. The Ti–EH sample had both ethanol and F127 non-ionic co-polymer and produced a mixture of anatase, rutile, and brookite phases. The amount of water and type of alcohol affect the amount of residual HCl present in the reaction system. The aging step causes the rapid evaporation of ethanol and water and this leads to increased acidity. An increased acidity favors the transformation of anatase to rutile. Longer chain alcohols such as 1-butanol or 1-octanol produced pure rutile TiO_2_ mesoporous materials using the EISA approach. As seen in Figure 9, the four samples in the wide-angle powder XRD analysis show high crystallinity. However, in many mesoporous TiO_2_ materials, the level of crystallinity is low as noted by the broadness of the peaks and fewer reflections at higher angles.

**Figure 10.**
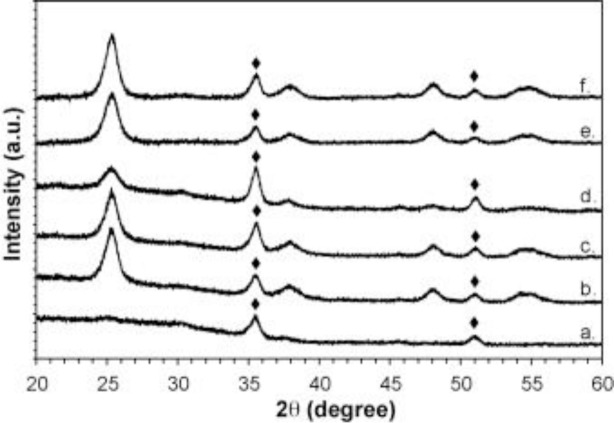
The XRD diffraction patterns reveal the level of crystallinity of the anatase TiO_2_ mesoporous thin films as function of relative humidity and calcination temperature: (a) RC-350; (b) R10-350; (c) R40-350; (d) R80-350; (e) R80-450; and (f) R80-550, where the number after R represents Relative Humidity and the number after hyphen is the calcination temperature. Reprinted with permission from [77]. Copyright 2009 Elsevier.

Figure 10 shows the wide-angle powder XRD of mesoporous thin films as function of humidity and calcination temperatures [77]. Mesoporous TiO_2_ films were made by taking TiO_2_ nanoparticles and combining with F127/acac/HCl/H_2_O/EtOH to form a sol. The sol was dip-coated. The RH value was varied from 10 to 80% in a humidity chamber. The films were heated at 40 °C for 48 h followed by drying at 110 °C for 24 h in air. Then, the crystallinity of the films were altered by varying the calcination thermal treatments for 4 h at 350, 450, or 550 °C. The sample labeled (a) had the least crystallinity. As the RH value was increased to 10, 40, and 80% respectively, the crystallinity seems to vary as seen from the XRD plots for samples labeled as (b), (c), and (d), respectively. All these samples were calcined at 350 °C. The sample labeled (f) was prepared with 80% RH and calcined at 550 °C and this sample had crystallinity similar to sample (b). Finally, in this section, we see that RH and calcination temperature affects the crystallinity of the material the most. Other variables such as aging time, nature of solvent, and surfactant also influence the phase of TiO_2_ [46,77].

### 3.2. Kr and N_2_ Physisorption

**Figure 11.**
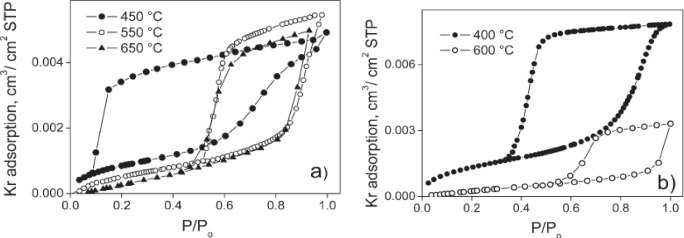
Kr physisorption as function of (**a**) calcination temperature and (**b**) type of non-ionic surfactant employed to produce TiO_2_ films. Reprinted with permission from [54]. Copyright 2007 John Wiley and Sons.

The textural properties of the mesoporous materials are determined by physisorption of gases such as N_2_. In recent years, Ar and Kr gases have also been used for the analysis of textural properties. The Kr physisorption analysis is similar to nitrogen physisorption with the exception that the vapor pressure of Kr is lower compared to N_2_ at 77 K and 1 atm. Kr gas is usually employed for thin films [108]. Figure 11 shows the Kr adsorption isotherms of mesoporous TiO_2_ thin films at 77 K made with two different non-ionic copolymer surfactants, KLE and Pluronic P123 [54]. In the figure on the left, the adsorption of Kr on TiO_2_ films calcined at three different temperatures is plotted. The hysteresis loops show variations as the calcination temperature is changed. In particular, the material calcined at 450 °C show a different isotherm compared to the other two samples. This thin film shows pore blocking phenomenon in which the pores are emptied at very low relative pressures. This is typical of materials that have ink-bottle type of pores. However, the surface area is the largest for the sample calcined at 450 °C. The sample calcined at 550 °C has enhanced pore volume value compared to either 450 °C or 650 °C samples, and this result could be expected from less pore blockage and pore contraction. The material calcined at 650 °C has slightly larger pore volume than the one calcined at 450 °C from less pore blockage. The sample calcined at 650 °C has the least surface area and largest pore diameter compared to the rest of the samples. The parts of the Kr-isotherm can be broken into three sections of relative pressure values of (*p*/*p*_0_): (1) 0.0–0.3; (2) 0.3–0.6; and (3) 0.6–1.0. Section 1 is related to monolayer adsorption of Kr in the pores of the material. Section 2 is multilayer adsorption, and Section 3 is due to capillary condensation [108]. In Figure 11b, the isotherm of mesoporous TiO_2_ thin films prepared using P123 and calcined at two different temperatures is shown. The sample calcined at 400 °C shows a very steep decrease during the desorption process and this is indicative of narrow pore size distribution. The sample calcined at 600 °C shows lower amounts of adsorption of Kr, indicating lower surface area and pore volume.

**Figure 12.**
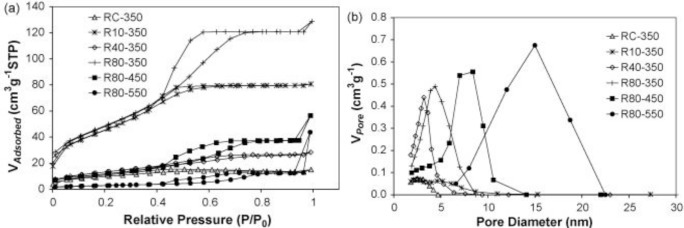
Nitrogen isotherms of mesoporous titania films (**a**) effect of calcination temperatures; and (**b**) corresponding pore-size-distribution plots. Reprinted with permission from [77]. 2009 Elsevier.

Nitrogen physisorption analysis is also commonly employed for evaluating the textural properties. Zhao and co-workers used EISA method to prepare mesoporous TiO_2_ films and these materials had different textural properties as a function of humidity and calcination conditions as the isotherm and pore size distribution plots in Figure 12 indicate [77].

Figure 12a shows the various isotherms when RH in the aging step and the final calcination thermal treatment are varied. The sample calcined at 350 °C has the largest surface area and pore volume values, and the sample calcined at 550 °C has the least specific surface area. The nitrogen isotherms appear to be type IV that is typical of mesoporous materials with a type H2 hysteresis loop indicating presence of pores with narrow mouths [5,107,111]. The pore size distribution plots shown in Figure 12b become progressively larger with higher calcination temperatures, which support the claim of lower surface area and pore volume values as indicated in Figure 12a [77]. Overall, the results in the nitrogen physisorption analysis point to the modulation of textural properties brought by the varying the calcination temperature.

### 3.3. Raman Spectroscopy

**Figure 13.**
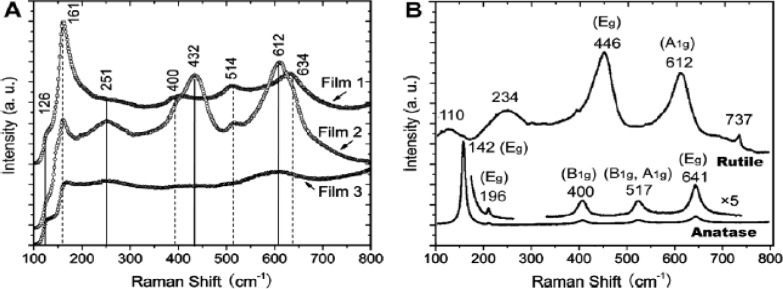
Raman spectra of (**A**) different TiO_2_ mesoporous films and (**B**) reference anatase and rutile. Reprinted with permission from [47]. Copyright 2008 Elsevier.

Raman spectroscopy provides information regarding the type of TiO_2_ phase and the level of crystallinity [26,29]. In addition, the presence of residual surfactant remnants can also be discerned from Raman studies. Figure 13A shows the Raman spectra of three TiO_2_ mesoporous films [47]. From comparing the Raman spectra of reference anatase and rutile films in Figure 13B, Film 1 contain bands at 161 (E_g_), 400 (B_1g_), 514 (B_1g_ and A_1g_), and 634 cm^−1^ (E_g_) from the anatase phase of the mesoporous TiO_2_ film. Film 2 contains both anatase and rutile TiO_2_ phases with bands seen at 126 (B_1g_), 251 (E_g_), 432 (E_g_), and 612 cm^−1^ (A_1g_) from rutile in addition to the bands due to anatase phases. Film 3 also has a combination of both anatase and rutile phases. The Raman peak positions is function of the particular symmetry mode, which have been noted previously [112]. These Raman peak shifts provide a context for identifying the crystallographic structure and phase, such as in TiO_2_ [113,114]. The basis for Raman shift is found in forming instantaneous induced dipole moments, and this dipole moment is function of the corresponding resulting electric field (E) and polarizability tensor (α). The product of α × E gives the instantaneous induced dipole moment (μ_inst_). However, the fundamental basis of μ_inst_ is group theory applied to solids, such as TiO_2_. Group theory provides an avenue to delineate the Raman active modes from use of symmetry tables and related character tables. Raman band positions depend on the type of TiO_2_ phase; therefore, these peak positions act as signature of a particular phase [115]. Both the anatase and rutile phases have 6 atoms per unit cell and hence a total of 3N-3 vibrations are theoretically possible. However, for anatase, only the modes, A_1g_, B_1g_, and E_g_ are active [116]. Similarly, the rutile phase is comprised of four Raman active modes (A_1g_, B_1g_, B_2g_, and E_g_), which includes both bending and stretching vibrations [117]. The Raman analysis is semi-quantitative in nature because the absolute values for the cross-sectional absorption is difficult to be computed exactly.

### 3.4. X-ray Scattering

**Figure 14.**
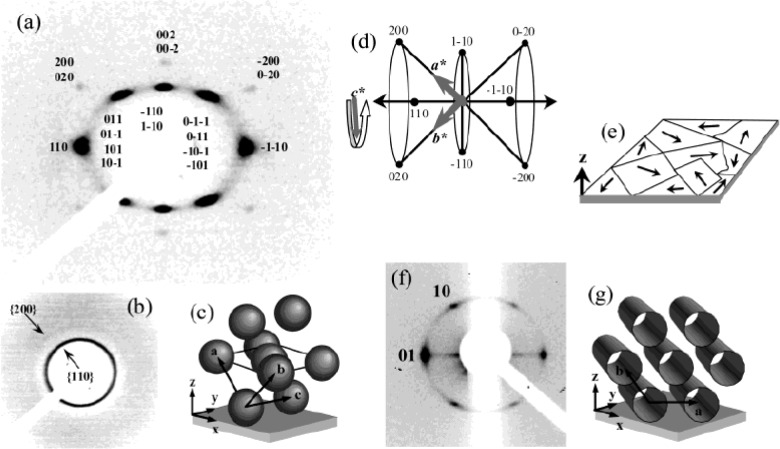
(**a**) Small Angle X-ray Scattering (SAXS) pattern collected at incident angles of (**a**) 4°; (**b**) 90° of a cubic TiO_2_ film; (**c**) representation of a cubic domain; (**d**) reciprocal space; (**e**) representation of film orientation; (**f**) SAXS pattern of a hexagonal film; and (**g**) alignment of 2-D domains. Reprinted with permission from [62]. Copyright 2003 American Chemical Society.

X-ray scattering is a non-destructive analysis that provides information regarding chemical composition and type of crystal structure [110,118,119]. Materials that are partially ordered and lack sufficient crystallinity are difficult to be characterized fully by XRD and in this respect, Small Angle X-ray Scattering (SAXS) is a powerful technique that provides rich information for mesostructured materials. Figure 14a,b show the indexed SAXS patterns collected at two different incident angles (4° and 90° respectively) for mesoporous titania thin films prepared using using F127 surfactant and this pattern is typical for a cubic symmetry structure [62]. The cubic domain is represented in Figure 14c. A family of planes are indicated in the reciprocal space map in Figure 14d,e provides a picture of orientation of the planes with the z axis normal to the substrate. Figure 14f shows the indexed SAXS pattern for hexagonal p6m symmetry structure, and the resulting picture of how the hexagonal structures are aligned is shown in Figure 14g. In comparing the SAXS patterns, shown in Figure 14a,f, one can see that the (01) planes are aligned to parallel to the surface in the hexagonal films whereas the c axis has no preferred orientation. SAXS is a powerful technique to identify the crystal structure, phase, and space group of thin films such as TiO_2_.

### 3.5. Transmission Electron Microscopy (TEM)

**Figure 15.**
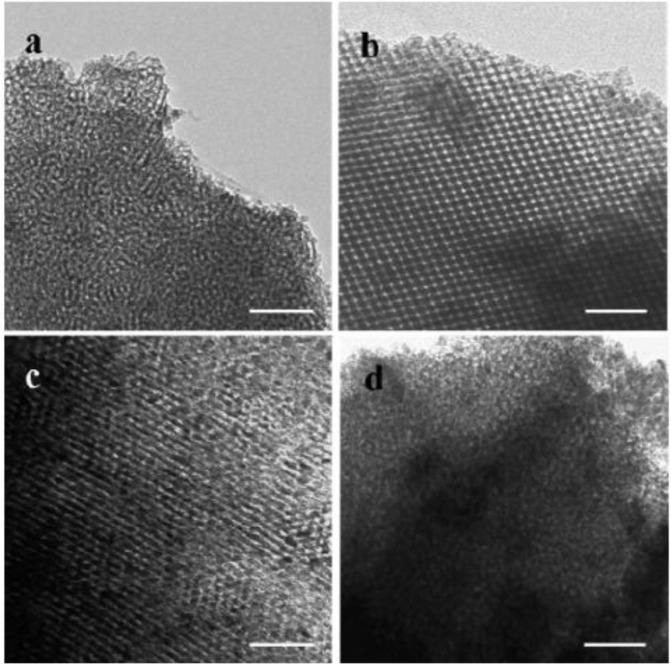
TEM images of TiO_2_ powders prepared with various TiCl_4_/Ti(OBu)_4_ ratios of (**a**) 2.5/0; (**b**) 2.0/0.5; (**c**) 1.0/1.5; and (**d**) 0/2.5. Scale bar is 50 nm. Reprinted with permission from [84]. Copyright 2007 American Chemical Society.

Transmission electron microscopy (TEM) analysis provides information regarding morphology and degree of pore ordering. Figure 15 show TEM images of TiO_2_ powders prepared using different ratios of TiCl_4_/Ti(OBu)_4_ [84]. In Figure 15a, the TEM of TiO_2_ prepared using TiCl_4_ is shown and the structure appears to have wormhole like structure with very little pore ordering. The sample prepared with ratio of 2.0 TiCl_4_/0.5 Ti(OBu)_4_ leads to hexagonal ordered pore structure as shown in Figure 15b. However, at molar ratios of 1.0 TiCl_4_/1.5 Ti(OBu)_4_ lower pore ordering is seen in Figure 15c. The trend of lower pore ordering continues with Figure 15d showing essentially little pore ordering similar to wormhole structure in Figure 15a. The molar ratio in Figure 15d is 0 TiCl_4_/2.5 Ti(OBu)_4_. These results confirm the need for careful modulation of the acidity from HCl (formed by the hydrolysis of TiCl_4_). Too much or too little acid content leads to poorly formed mesoporous TiO_2_ structures.

**Figure 16.**
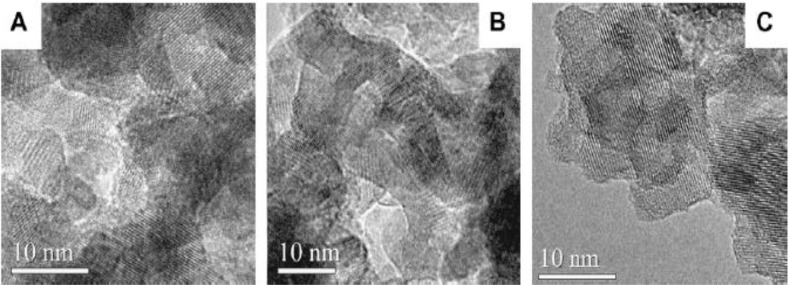
High Resolution TEM (HRTEM) images of three mesoporous TiO_2_ films. (**A**) Film 1; (**B**) Film 2; and (**C**) Film 3. Scale bar is 10 nm. Reprinted with permission from [47]. Copyright 2008 Elsevier.

Similar to conventional TEM analysis, High Resolution TEM (HRTEM) provides information on the *d*-spacings from the lattice fringes present and helps in identifying the phase(s) of TiO_2_. In addition, the crystallite size of respective phases can be estimated and thus HRTEM studies complement powder XRD studies. Figure 16A–C shows three mesoporous TiO_2_ films that have many lattice fringes, thereby inferring that they are highly crystalline materials [47]. The crystallite size ranges from 4 to 12 nm in these materials. The crystallite size was constrained by calcining in two steps. The multi-step calcination process has been attempted by other researchers in forming mesoporous TiO_2_ powders and films [41,43,54,62,65,87,93,120–124]. The major challenge is in preserving the ordered mesoporous pore structure while maintaining crystallinity. Overall, TEM analysis helps in understanding the periodicity of the pores and HRTEM analysis helps in calculating the crystallite size and in identifying the phase(s) of titania. It should be mentioned that a key aspect of obtaining good quality TEM images lies in sample preparation. An extremely dilute suspension of the mesoporous material should be prepared to obtain good quality TEM. An alternative would be to ultramicrotome the mesoporous films if they were thick into thin slices in the 50–100 nm range.

### 3.6. Scanning Electron Microscopy (SEM)

**Figure 17.**
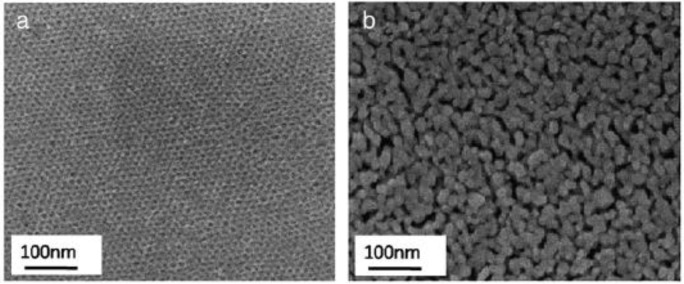
Depiction of mesoporous TiO_2_ films calcined at two different temperatures: (**a**) 430 °C and (**b**) 550 °C. Reprinted with permission from [91]. Copyright 2009 Elsevier.

Scanning Electron Microscopy (SEM) provides information regarding morphology and an overall picture of the ordering of the pores in porous materials. An advantage of using SEM is that sample preparation is very simple compared to TEM. Figure 17a,b show the changes in the ordering of the pore structure after calcination at 430 °C and 550 °C [91]. The pores appear to be arranged in a hexagonal geometry in Figure 17a. However, after calcination for 15 min. at 550 °C, the structure appears disorganized with the ordered pore channels destroyed. The TiO_2_ pore walls have collapsed into larger particles. This order-to-disorder transition has been observed by SEM by other researchers when samples have been calcined at temperatures higher than or equal to 400 °C [42,45,50,52,87,90,104,125,126]. Fu and co-workers [39] performed SEM analysis of the mesoporous TiO_2_ materials and observed agglomerated particles even when the sample was calcined at 350 °C. In contrast, other researchers have been able to design synthesis and thermal treatments to preserve the periodicity of the pores after calcination at temperatures of 400 °C or greater [44,47,54,55,124,127–129].

The central theme for producing mesoporous TiO_2_ materials that are ordered after calcination at 400 °C and higher temperatures seems to be the need for performing extensive pretreatment steps in conjunction with multiple step thermal treatment.

### 3.7. Atomic Force Microscopy (AFM)

**Figure 18.**
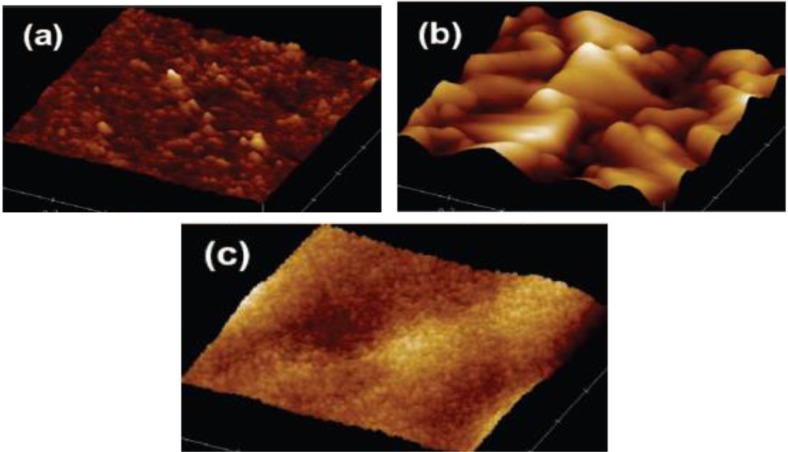
AFM images of (**a**) bare Pyrex glass; (**b**) FTO layer; and (**c**) Meso-TiO_2_/FTO layer. Reprinted with permission from [48]. Copyright 2008 American Chemical Society.

Similar to TEM and SEM analysis, AFM provides surface characteristics of a material from the atomic force interactions between the probe and the surface. The various modes of atomic force microscopy permit determining surface roughness, surface porosity, and particle size [130]. AFM is completed in either equilibrium, tapping, or contact mode. In equilibrium mode, the tip is kept a pre-determined distance from the substrate and moved across in a scanning motion. The valence electron shells are probed in this mode, thereby providing a mapping of the surface atoms from the slight defection in the cantilever arm attached to the tip. The tapping mode provides information on the surface topology from the repulsive force as the tip contacts the surface. This gives a defection measured by the laser beam reflected onto the photo detector as a voltage signal. The contact or static mode involves placing the tip on the surface of the substrate and mechanically dragging the tip. The force between the tip and the surface is balanced by the elastic force produced by the deflected cantilever, and this provides information on the surface roughness. The contact mode can be carried out in two ways, at constant force or constant height. The advantage in the contact mode is that atomic resolution can be obtained and high scanning speeds can be achieved. The greatest spatial resolution is obtained with sharp tips. Nevertheless, the tip must be carefully chosen depending on the information desired from the AFM analysis, such as porosity and roughness. Figure 18a–c show the morphologies of different substrates and that of TiO_2_ film, which was completed using AFM in the contact mode and also known as Lateral Force Microscopy (LFM) [48]. The AFM of Pyrex glass as shown in Figure 18a has a roughness value of 0.9 nm; in contrast, the FTO layer as shown in Figure 18b has peak height of 34 nm. These values illustrate the ability to differentiate surface topology by simply dragging the tip across the substrate. Application of mesoporous TiO_2_ on the FTO substrate appears to reduce the roughness as seen in Figure 18c.

Similar results with mesoporous TiO_2_ materials are also seen in other works [36,128]. Zhang and Wang observed highly ordered pore arrangements of TiO_2_ from AFM analysis [128]. However, they did not show AFM analysis results of the calcined TiO_2_ materials. In summary, AFM analysis of mesoporous TiO_2_ provides important information on the thickness of the TiO_2_ film and surface pore ordering.

### 3.8. X-ray Photoelectron Spectroscopy (XPS)

**Figure 19.**
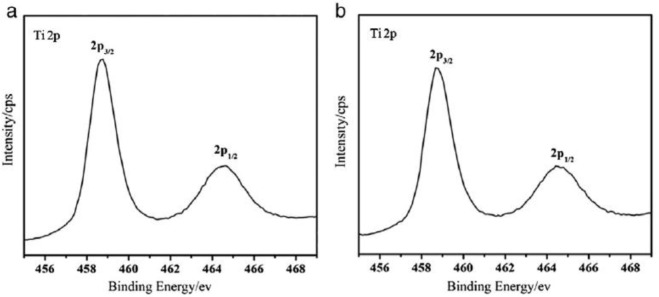

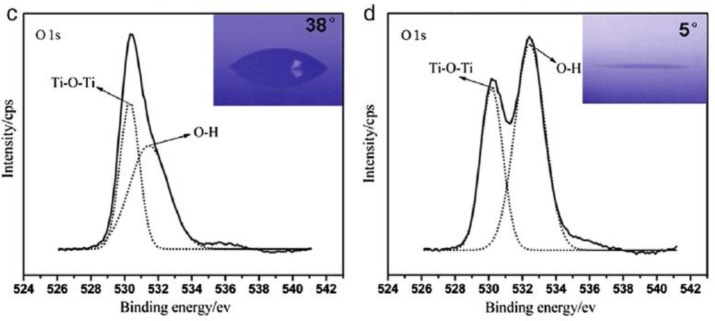
X-ray Photoelectron Spectroscopy (XPS) spectra of the Ti 2p region of mesoporous TiO_2_ film calcined at (**a**) 350 °C; (**b**) 450 °C; O 1s spectra of TiO_2_ film calcined at (**c**) 350 °C; and (**d**) 450 °C. Inset shows the contact angles. Reprinted with permission from [44]. Copyright 2012 Elsevier.

X-ray Photoelectron Spectroscopy (XPS) is a surface sensitive technique for determining the composition, chemical and electronic state(s) of elements and it can also be used to detect the presence of impurities and types of surface groups [131,132]. The oxidation states of atoms on the surface of a given material can be differentiated by their binding energies. The binding energy is a measure of the energy needed to remove a core electron from an atom. XPS has been used to investigate the presence of residual impurities (carbonaceous and nitrogenous) in calcined mesoporous TiO_2_ materials. Fu and co-workers [43] found that their mesoporous TiO_2_ powdered samples still had residual nitrogen fragments due to en chelating agent from the N 1s peak present at 399.5 eV even after calcination at 500 °C and 600 °C. In contrast, Wang and co-workers [133] found that their mesoporous TiO_2_ thin films calcined at 500 °C had essentially no carbon residue from their XPS analysis. Wang and co-workers [102] discovered that their mesoporous TiO_2_ thin film had carbon residue in the form of carboxyl group from a peak at 288.3 eV in the C 1s spectrum. The carbon C 1s standard is 284.4 ± 0.2 eV [131]. Shifts in the binding energy value indicate changes in the chemical environment. In addition, these researchers noted that the O 1s spectrum had two types of oxygen species with peaks at 531.5 and 529.9 eV due to surface hydroxyl groups and lattice oxygen (Ti–O–Ti bonds) [102]. The Ti 2p XPS analysis of mesoporous TiO_2_ materials show two peaks at approximately 464.2 and 458.5 eV, which are from Ti 2p_3/2_ and Ti 2p_1/2_ peaks [44,92,102]. Figure 19a,d shows the Ti 2p and O 1s spectra as function of calcination temperature [44]. The XPS results are also presented as function of the degree of hydrophobicity in Figure 19c,d. In Figure 19a,b, the Ti 2p spectra show little to no peak shift with changes in the calcination temperature from 350 °C to 450 °C. The peak positions in Ti 2p spectrum indicate presence of Ti^4+^. The 530.2 eV peak in the O 1s spectrum is related to the Ti–O–Ti bonding in TiO_2_ as shown in Figure 19c,d. The intensity of the peak at 532.4 eV increases upon calcination from 350 °C to 450 °C in the O 1s spectrum in Figure 19c,d, indicating an increase in the presence of hydroxyl groups [44,134–136].

### 3.9. Diffuse Reflectance Spectroscopy (DRS)

Diffuse Reflectance Spectroscopy (DRS) of mesoporous TiO_2_ provides information on the bandgap, particle size, and phase. Since, titania (anatase phase), absorbs only in the UV region, the wavelength region of 320 to 380 nm is of importance for estimation of bandgap and particle size of anatase. The rutile phase of titania has a lower bandgap and the onset of absorption is shifted to the visible region, *i.e*., near 400 nm. The bandgap of titania is calculated from the DRS spectra by extrapolating the high slope region to the *X*-axis. Shifts in onset of absorption indicate changes in the particle size corresponding to various band gaps. The particle diameter (R) of titania can be calculated using the Brus formula, ΔE_g_ = (ħ^2^π^2^/2R^2^μ) − 1.8e^2^/εR [137]. ΔE_g_ is the difference in the band gap between bulk titania (anatase with a band gap of 3.2 eV or rutile with a band gap of 3.0 eV) and the band gap of titania prepared in the study. The other constants in the above equation are: ħ = reduced Planck’s constant, μ = reduced mass of the exciton, *i.e*., 1/μ = 1/m^*^_e_ + 1/m^*^_h_ where m^*^_e_, is the effective mass of the electron in TiO_2_, and m^*^_h_ is the effective mass of the hole in TiO_2_. A value of m^*^_e_ = 9 m_e_ and m^*^_h_ = 2 m_e_ is typically used, where m_e_ is the mass of electron, and ε is the dielectric constant of titania = 184. Yu and co-workers [138] made several mesoporous TiO_2_ thin films, and they characterized the films by DRS, which showed a change in the peak onset for each of the films, indicating a change in band gap and the particle size of TiO_2_. Likewise, Dai and co-workers [64] were able to determine the particle size of TiO_2_ thin films using DRS. Several other works employed DRS to determine band gap and particle size for TiO_2_ prepared by the EISA method [38,45,52,129,139]. The research works taken together points to the versatility of DRS for obtaining the type of crystallographic phase, bandgap, and particle size.

### 3.10. Fourier Transform-InfraRed (FT-IR) Spectroscopy

**Figure 20.**
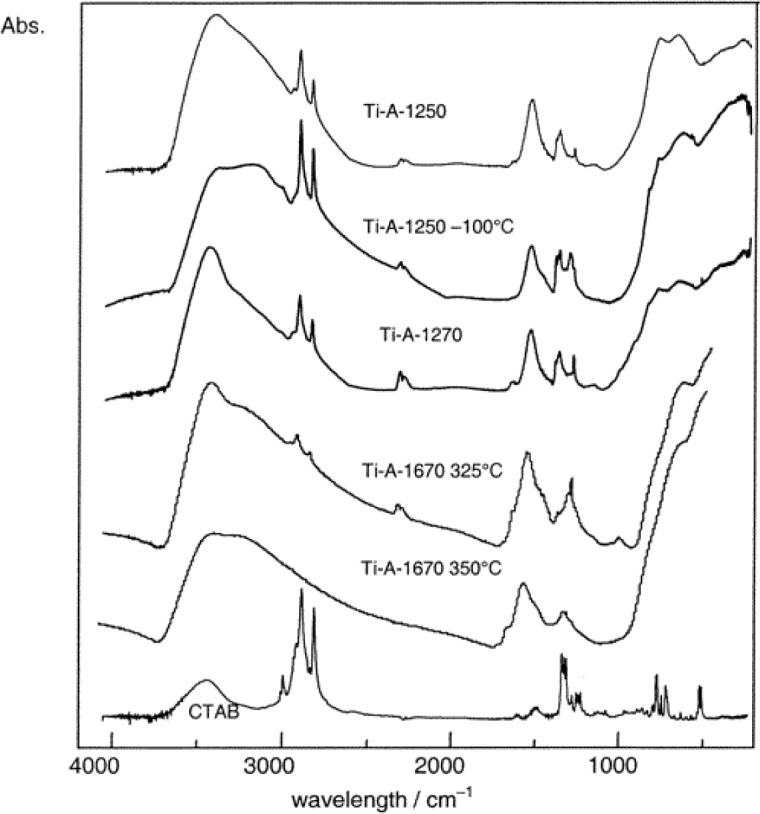
FT-IR spectra of mesoporous TiO_2_ hybrid solids prepared using CTAB. Reprinted with permission from [83]. Copyright 2002 American Chemical Society.

The main reason for using FT-IR in characterization mesoporous TiO_2_ materials is for determining if residual surfactant fragments are present after calcination treatment. Agarwala and Ho used FT-IR analysis and their studies revealed that the P123 non-ionic surfactant were present in the as-synthesized film from the bands present in the region 1025–1250 cm^−1^, and at 1714, 2877, 2969, and 3300 cm^−1^ [91]. These bands are from the surfactant due to C-O stretching band, vibrations of –CH_3_ groups, symmetrical and asymmetrical –CH_2_ stretchings, and –C–H vibrations respectively. In addition, a band at 1081 cm^−1^ band was observed, and this is due to partially condensed Ti–O linkages. However, after calcination at 430 °C, the bands at 1714 and 3300 cm^−1^ corresponding to –CH_3_ and –CH vibrations reduced dramatically. Therefore, FT-IR analysis provides evidence for partial removal of the non-ionic surfactant after calcination. Bhaumik and co-workers also observed alkyl stretchings at 2920 and 2851 cm^−1^, which disappear upon calcination and with simultaneous introduction of a broad peak in the 3200–3470 cm^−1^ region due to surface hydroxyl groups in their mesoporous powders [52]. In contrast to the bands previously discussed, Fu and co-workers discovered a band at 3420 cm^−1^ in their mesoporous TiO_2_ powders, which they attribute to the presence of N–H fragment from the use of en as a chelating agent [43]. This N–H fragment is retained until 800 °C, and when the fragment is eliminated at 800 °C, the structure collapses. Figure 20 shows the FT-IR spectra of mesoporous TiO_2_ solids. Bands due to surface hydroxyl groups and strongly bound water molecules (in the range of 4000–3000 cm^−1^), stretching bands due to –CH from methylene and methyl groups (3000–2800 cm^−1^), bending vibrations due OH, CH, CO respectively (1700–1000 cm^−1^), and stretching vibrations due to Ti–O–Ti (900–400 cm^−1^) are seen [83]. After calcination at 350 °C, bands in the 3000–2800 cm^−1^ region do not seem to be present. This material, however, has peaks in the 1700–1000 cm^−1^ region, due to water and CTAB fragments. The shoulder bands below 900 cm^−1^ are from Ti–O–Ti linkages. Thus, the FT-IR results of this hybrid TiO_2_ material shows some residual carbon fragments from the cationic surfactant after thermal calcination treatment.

**Figure 21.**
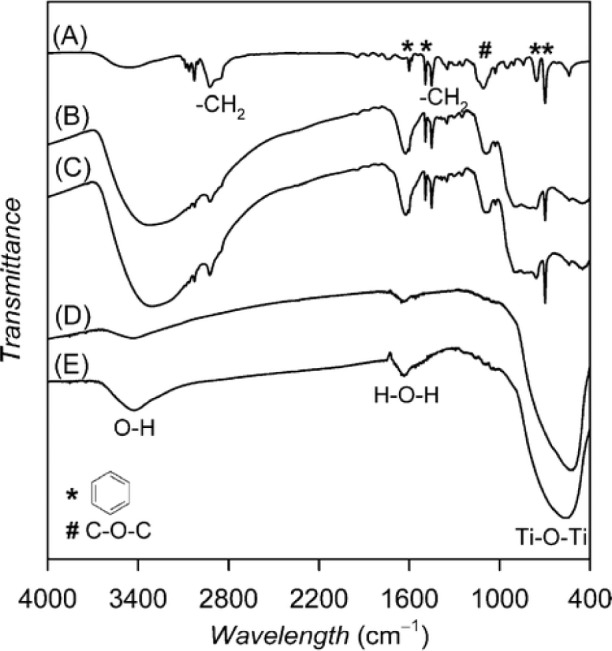
FT-IR spectra of non-ionic prepared mesoporous TiO_2_ hybrid thin films. Reprinted with permission from [133]. Copyright 2011 American Chemical Society.

Figure 21 shows the FT-IR analysis of mesoporous TiO_2_ hybrid thin films prepared using non-ionic surfactant [133]. The PS-*b*-PEO non-ionic copolymer has bands attributed to benzene ring at 700, 760, 1490 and 1600 cm^−1^. In addition, there is a band at 1115 cm^−1^ from C–O–C stretching from PS and PEO portions of the block copolymer. The bands in the regions of 3000–2800 and 1485–1330 cm^−1^ are from CH stretching and bending modes in –CH_2_. The bands at 3350 cm^−1^ and 1640 cm^−1^ band are due to stretching vibrations from water. The band near 400 cm^−1^ is from Ti–O–Ti framework, which increases upon calcination at 500 °C. In summary, FT-IR is useful to deduce the presence of residual surfactants in the calcined materials.

### 3.11. Thermal Gravimetric Analysis (TGA) and Differential Scanning Calorimetry (DSC)

**Figure 22.**
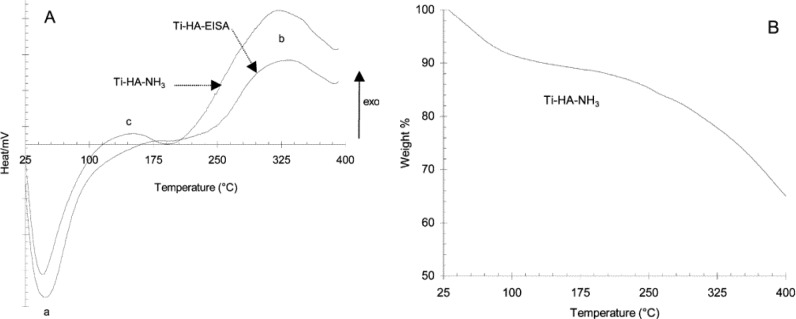
Differential Scanning Calorimetry (DSC) curve in part (**A**) and Thermo-Gravimetric Analysis (TGA) curve in part (**B**) of mesoporous TiO_2_ powders treated with NH_3_. Reprinted with permission from [98]. Copyright 2004 American Chemical Society.

Thermal gravimetric analysis (TGA) provides information regarding the minimum temperature required to remove the surfactant molecules from the pores. In addition, the weight losses provide information regarding the nature of species eliminated during the calcination step. Differential scanning calorimetry (DSC) provides information regarding phase transitions. In mesoporous TiO_2_ solids prepared using cationic CTAB surfactant, Sanchez and co-workers noted the following temperature ranges and types of species present in the TGA experiments [83]. The weight loss at temperature below 100 °C is from loss of volatile species, such as water, ethanol, and HCl. A weight loss of 5–10 wt% in the 100–210 °C was observed and this was due to the start of the breakdown of the cationic surfactant. At 250 °C and 280 °C, the organic backbone of CTAB fragments into small carbon residues. Beyers and co-workers [100] also find a major weight loss at 270 °C similar to those previously observed by Sanchez and co-workers in their preparation of mesoporous powders [83]. However, if there was a NH_4_OH post-treatment step, then the removal of CTAB occurred slowly over a wider range of temperature as demonstrated in the work by Beyers and co-workers [100]. Ozin and co-workers [35] found mesoporous TiO_2_ thin films prepared with non-ionic P123 show a weight loss of 14.6% below 120 °C due to elimination of water, butanol, and HCl. Upon heating further from 120 °C to 280 °C, there is a weight loss of 36.5% from the decomposition of P123 non-ionic surfactant. Carbon residue from the decomposed P123 surfactant leaves the mesoporous TiO_2_ material from 280 °C to 380 °C with a weight loss of 5.67%. There is no weight loss upon heating to temperatures greater than 380 °C when P123 is used in the preparation of mesoporous TiO_2_ materials. Hongo and Yamazaki [40] also saw a similar TGA pattern from their mesoporous powder made with P123. In contrast, Fu and co-workers [43] had a different TGA result because of the use of en in their synthesis. Nitrogen containing fragments from en were completely eliminated from the mesoporous powders at calcination temperatures greater than 400 °C. The non-ionic surfactant, however, decomposed below 400 °C. Other researchers also discovered that non-ionic surfactants F127 and Brij 58 show similar TGA patterns as that of P123 with the surfactants decomposing below 400 °C [52,101]. Cassiers and co-workers employed a neutral amine template and found that the post-treatment procedure affected the resulting DSC/TGA curves, as shown in Figure 26 [98]. Figure 22A, shows the DSC curves of mesoporous powders prepared with and without NH_3_ treatment. Both the peaks in Figure 22A show removal of volatile compounds, such as water and ethanol, from 25 °C to 120 °C. This endothermic peak is related to a 10% decrease in the TGA result as shown in Figure 22B. A broad peak (labelled as b in part A) in the DSC experiments is seen with a weight loss of nearly 30% due to the simultaneous decomposition of the surfactant and crystallization of anatase TiO_2_ phase. The peak labelled as c in the DSC plot show a broad peak from 130 °C to 180 °C with a maximum at 160 °C, and this is attributed to an exothermic process that is due to the conversion of amorphous TiO_2_ transforming to crystalline rutile TiO_2_ phase. It should be noted that there is no weight loss at this temperature range in the TGA analysis, which further confirms the crystallization of the rutile phase. Finally, Carreon and co-workers noted that mesoporous TiO_2_ powder prepared with cationic CTAB had ~1.5% carbon residue compared to 0.9% from non-ionic P123 surfactant after calcination at identical temperatures [125].

## 4. Applications

The EISA method for producing mesoporous TiO_2_ materials has several advantages compared to conventional sol-gel preparation method. EISA provides an opportunity to prepare porous TiO_2_ films under relatively mild conditions [26,28]. Titanium dioxide films can be made using EISA with four film forming methods: dip-coating, spin-coating, meniscus drop forming, and spray coating [26]. These four film forming methods using the EISA method, open many avenues for producing high-quality TiO_2_ porous films. Pan and Lee noted that the EISA method permits the ability to tune the porosity by adjusting the aging period and RH [28]. The textural properties of TiO_2_ prepared by using EISA method are higher or comparable to other methods such as hydrothermal synthesis and combinations thereof. In addition to tuning the textural properties, the EISA method is quite versatile since different phases of TiO_2_ can be obtained by merely changing either the solvent (alcohol), precursor, water, RH, pH, or the aging period.

The following sections outline a few of the many applications that TiO_2_ materials are used in recent literature, such as Dye Sensitized Solar Cells (DSSCs), photocatalytic degradation of dyes and organics, photocatalytic splitting of water, and batteries. The focus is on TiO_2_ (films and powders) prepared by the EISA approach and the advantages of using EISA will be exemplified in this section.

### 4.1. Dye Sensitized Solar Cells (DSSCs)

DSSCs represent one of the most researched applications for mesoporous TiO_2_ materials [140–142]. Traditionally, for DSSC applications, TiO_2_ powders are first made and then a colloidal suspension is prepared and a film of the titania paste is deposited onto a conducting substrate such as FTO glass by the doctor blade method. Problems in this approach are that the nanoparticles need to agglomerate when dispersed in a solvent but most importantly, since the FTO substrate is fairly rough, the titania paste that contains agglomerated particles cannot completely cover FTO. As a result of the formation of voids, and poor contact between titania and FTO, the electrolytes can directly contact the FTO electrode and decrease the open circuit voltage by back electron transfer reaction. Mesoporous TiO_2_ materials prepared by EISA method have uniform pores aligned periodically, and large pore structures with good pore connectivity, few grain boundaries, high surface area, and large pore volumes. These factors favor efficient adsorption of dye molecule, diffusion of electrolyte, and good electron transport in DSSCs leading to greater efficiencies.

**Figure 23.**
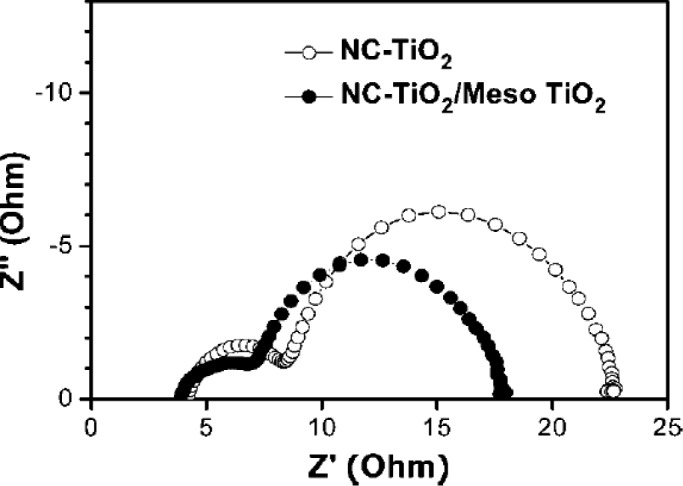
Electrochemical Impedance Spectroscopy of nanocrystalline TiO_2_ and EISA prepared mesoporous TiO_2_ with nanocrystalline TiO_2_. Reprinted with permission from [48]. Copyright 2008 American Chemical Society.

Lee and co-workers [48] employed EISA synthesis to produce cubic *Ia*3*m* mesoporous TiO_2_ thin films in DSSC application. These researchers used Electrochemical Impedance Spectroscopy (EIS) to determine the effects of depositing a thin layer of mesoporous TiO_2_ onto a layer of nanocrystalline TiO_2_. The Nyquist impedance plot of nanocrystalline (NC-TiO_2_) and NC-TiO_2_ with Meso-TiO_2_ is shown in Figure 23. The hemisphere is smaller for NC-TiO_2_-Meso-TiO_2_ suggesting that the resistance on the FTO-TiO_2_ interface is lower in this configuration. The result suggests that the Meso-TiO_2_ material provide a good contact between the FTO substrate and the nanocrystalline TiO_2_ layer. The photovoltaic conversion efficiency increased from 5.77% to 7.48% by use of a Meso-TiO_2_ interfacial layer, *i.e*., an improvement of 30%.

A combination of doctor blade technique and a two-step EISA method led to the formation of a crack-free mesoporous TiO_2_ film that had a solar conversion efficiency of 6.53% [89]. Zhao and co-workers [87] prepared a ligand-assisted mesoporous TiO_2_ film by the EISA method. These researchers fabricated a DSSC and compared the efficiencies of the mesoporous TiO_2_ material with a commercial Degussa P25. The power-conversion efficiencies were calculated to be 4.72 and 3.44% respectively. It was found that the mesoporous TiO_2_ film had higher surface area with fewer defects, adsorbed larger amounts of dye, and had longer electron life-times. These favorable properties led to higher Incident to Photon-Conversion Efficiency (IPCE). The power-conversion efficiency could be increased to as high as 5.45% by increasing the thickness of the mesoporous TiO_2_ film. A similar strategy was realized by depositing a mesoporous TiO_2_ thin film layer of 4 μm thickness and having a Degussa P25 TiO_2_ material as a top layer and such a configuration lead to a 200% increase in cell efficiency compared to the bare Degussa P25 [129]. An EISA method was used to prepare mesoporous TiO_2_ with three different pore diameters of 6.5, 8.2, and 11.0 nm. DSSC with the 8.2 nm pore size had the highest photoelectrical conversion efficiency in this study [39]. At larger pore sizes than those required for the optimal diffusion of the redox shuttle, charge-carrier recombination is favored and the efficiency drops. EISA method was used to prepare TiO_2_ nanocrystals inside titania nanotubes thin films and the resulting composite materials exhibited a 150% increase in light conversion efficiency compared to the bare nanotubes [143]. A multilayered mesoporous titania films was prepared using P123 polymer. Optically transparent and crack-free films were obtained. A film consisting of 8 layers and 1.9 μm thick was found to have a solar conversion efficiency of 4.63% [144]. In a related study phosphorus-modified mesoporous titania films of 1.8 μm thickness were sensitized with N-945 dye and a conversion efficiency of 5.03% was obtained [145]. Use of too much phosphorous lead to an anatase to TiO_2_(B) transition, which lowered the solar cell efficiency dramatically [146]. The solar cell efficiencies (η) varied widely for the mesoporous TiO_2_ materials depending on the type of fabrication. Innocenzi and co-workers prepared mesoporous TiO_2_ film by a dip-coating method. This film had a lower IPCE value compared to a standard TiO_2_ film that was prepared without pluronic P105 copolymer [120]. The mesoporous TiO_2_ film was only approximately 2 μm thick. In contrast, the standard TiO_2_ film was 7 μm thick and the lower efficiency was attributed to this factor. Weng and co-workers [89] employed a 7 μm thick mesoporous TiO_2_ film using the doctor blade method and obtained a 6.53% solar cell efficiency. Sung and co-workers [92] used EISA synthesis method to produce both mesoporous TiO_2_ and Al_2_O_3_ coated mesoporous TiO_2_ materials. The one-mole percent Al_2_O_3_ coated mesoporous TiO_2_ material had an efficiency of 6.50% compared to the pure mesoporous TiO_2_ material, which showed an efficiency of 5.88%. The higher efficiency appears to be linked to the presence of Al_2_O_3_ coating preventing back electron reaction. Eguchi and co-workers [147] discovered from EIS analysis that the TiO_2_ material must be calcined at minimally 400 °C to reduce grain boundaries, or the solar cell electron transfer was greatly reduced with the electrons accumulating at these junctions between the crystallites. This supports the argument that calcination at least 500 °C is necessary for good crystallinity of anatase and electron transfer properties. Free standing arrays of 1D titania nanowires, 3D gyroidal structured titania, and a disordered nanoparticle film were prepared and their solar cell performances were investigated. The efficiency of the gyroid films was found to be highest and this was attributed to the better structural integrity of the gyroid films although the 1D system exhibited enhanced charge transport. In a subsequent work, a bicontinuous free standing gyroid network of titania was prepared by a electrochemical method with 1.7% power conversion efficiency in spite of the relatively low thickness of the film (400 nm) [148]. Pluronic F127 was used to prepare a thick (5–6 μm) mesoporous titania films and efficiencies in the range of 6%–7% were reported [75].

In summary, the solar cell efficiencies of mesoporous TiO_2_ materials prepared by EISA depend on thickness and number of deposited layers, presence of blocking layers such as Al_2_O_3_, light scattering layers, and related textural properties such as pore architecture and size, and surface areas for a given dye molecule.

### 4.2. Photocatalytic Degradation of Organics

The photocatalytic degradation of organics have been explored extensively using mesoporous TiO_2_ materials because their large uniform pores and high surface areas provide efficient molecular trafficking for pollutants to enter and be converted to innocuous products.

**Figure 24.**
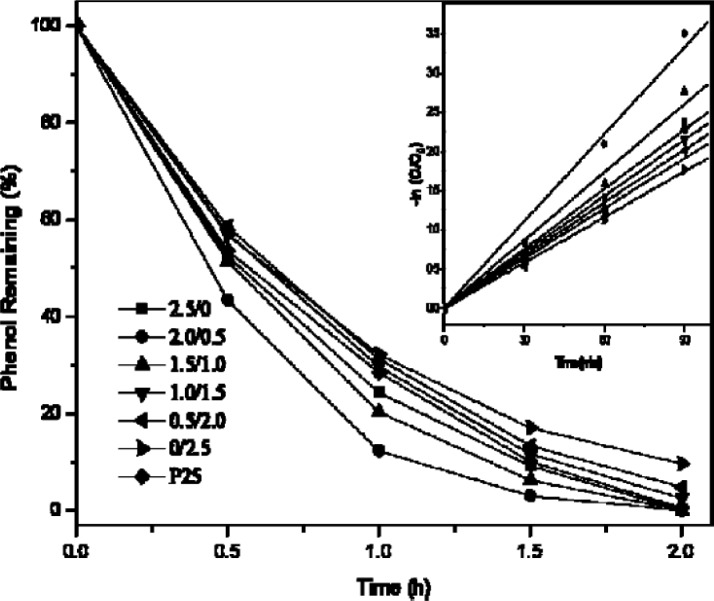
Phenol photodegradation using mesoporous TiO_2_. Reprinted with permission from [84]. 2007 American Chemical Society.

Mesoporous TiO_2_ powder was prepared using CTAB and a neutral surfactant (hexadecylamine) and the photocatalytic degradation of Rhodamine 6G was examined. An important observation was that the formation of the titania phase was affected by the choice of the surfactant and pH and this affected the photocatalytic performance [149]. A highly stable and crystalline mesoporous anatase powder was prepared by using a simple surfactant-sulfuric acid carbonization. This material exhibited higher photocatalytic activity for the degradation of Rhodamine B than a standard commercial sample, Degussa P25 [41]. The degradation of two dyes, Methylene Blue (MB) and Rose Bengal were investigated over TiO_2_ powders prepared using non-ionic surfactant, F127. Higher activity was observed for the mesoporous material compared to Degussa P25 [52]. Ayral and co-workers [104] studied the photodegradation of MB using mesoporous TiO_2_ films prepared by slip-casting and compared it with other TiO_2_ materials having both micropores and macropores. The TiO_2_ material that contained mainly mesopores degraded MB the most, compared to the other TiO_2_ materials having micropores and macropores. Non-ionic (P123) and cationic (CTAB) surfactants were used to prepare mesoporous anatase powders. The resulting TiO_2_ materials had surface areas of nearly 150 m^2^/g and highly uniform pores of 3 and 6 nm respectively. The UV degradation of MB, Methyl Orange (MO), Methyl Red, and Rhodamine 6G were examined and the mesoporous materials showed 2–3 times higher activity than a conventionally prepared anatase material without the surfactant. The higher activity for the mesoporous materials were attributed to the small crystallite size (~10 nm) and large pore sizes [125]. In another study, the decomposition of MO was found to follow Langmuir-Hinshelwood kinetics over mesoporous titania powders prepared using P123 surfactant [150]. The photocatalyst could be repeatedly used (four cycles) without any loss of activity. A mesoporous titania thin film prepared using non-ionic surfactant F127 and calcined at 500 °C degraded almost completely MO after 5 h of UV illumination [151]. The photoelectrocatalytic degradation rates for four dyes (MO, MB, Rhodamine B, and reactive brilliant red (K-2G)) were studied by Yuan and co-workers over titania nanotube film arrays containing TiO_2_ nanoparticles that were prepared by the EISA method [152]. The surface area of the nanotube array was found to increase by the deposition of the titania nanoparticles. Most importantly, the width of the space charge layer was also enhanced by this treatment procedure; this promoted the separation of the electron-hole pairs, and resulted in higher photocatalytic activities for the nanoparticle containing nanotubes in comparison to the nanotubes alone. Similarly, Wang and co-workers [151] noted anatase crystallite size, pore size, and surface area were major factors in the MB photodegradation rate using mesoporous thin films of titania. Even though calcination at 500 °C led to reduced textural properties, the formation of a highly crystalline TiO_2_ phase was found to be very important in these mesoporous TiO_2_ materials prepared using EISA approach. The photocatalytic activity of mesoporous titania powders prepared by EISA was compared with Degussa P25 in both gas and aqueous phases. In the gas phase degradation of toluene, the photocatalytic activity of mesoporous titania was higher in comparison to that of P25. However, in the aqueous phase degradation of Rhodamine 6G, the P25 material exhibited superior activity due to enhanced diffusion kinetics [99]. Post-modification of titania with NaOH led to a mesoporous material with higher surface area and larger pore volume than that compared to the use of NH_4_OH; in turn the photocatalytic activity for degradation of Rhodamine 6G was higher when mesoporous TiO_2_ powder was treated with NaOH [100]. Higher surface areas, homogeneous and uniform pores (11 nm), and high crystallinity were factors that enhanced the activity of mesoporous TiO_2_ powders for the gas phase degradation of acetaldehyde [138]. The phase of mesoporous TiO_2_ powders can be varied by using the EISA synthesis approach [46]. Chen and co-workers [84] were able to synthesize EISA prepared mesoporous TiO_2_ powders with varied anatase and rutile content by changing the TiCl_4_/Ti(OBu)_4_ molar ratio, and the photodegradation of phenol was studied as shown in Figure 24. When no TiCl_4_ was employed in the synthesis of mesoporous TiO_2_, pure anatase was produced, and this had the lowest activity for photodegradation of phenol. The use of TiCl_4_ led to the formation of a rutile phase, whereas using both TiCl_4_ and Ti(OBu)_4_ give a mixture of anatase and rutile phases. The use of a molar ratio of 2.0 TiCl_4_/0.5 Ti(OBu)_4_ produced a mixed phase of titania and this showed the highest activity for phenol photodegradation, which was noted to be from the efficient charge separation of the electron-hole pairs due to the presence of anatase and rutile phases.

Thermally stable mesoporous TiO_2_ powder was employed for the degradation of 2,4-dichlorophenol and it showed higher activity compared to Degussa P25 [43]. The higher activity was attributed to high crystallinity, high surface area, and large pore sizes. Surface photovoltage spectroscopic studies indicated lower recombination of electron-hole pairs in the mesoporous material. A surfactant and liquid crystal templating processes were combined to produce a highly ordered and semi-crystalline mesoporous titania photocatalyst powders. This material degraded 96% of MO dye after 3 h of irradiation [96]. In acetic acid photodegradation using mesoporous TiO_2_ powders, Hongo and Yamazaki [40] found that calcination at 400 °C gave the highest activity compared with other calcination temperatures, such as 350 °C and 450 °C. These researchers attribute the higher photodegradation rate of acetic acid on the enhanced textural properties, such as pore diameter and relatively good TiO_2_ crystallinity after calcination at 400 °C. A mesoporous thin film was examined for degradation of methyl stearate and complete degradation was achieved after 60 h at very low intensity of incident radiation, 1 mW/cm^2^. In contrast, a non-porous sample required twice as long. The mesoporous film was found to be super hydrophilic, with a contact angle of 5° (for water droplets) after UV illumination and remained sufficiently hydrophilic even in the dark [153]. The need for a compromise between crystallinity of TiO_2_, pore order, and textural properties appears to be important in the photodegradation of organics. In a recent study, eight photocatalysts including some commercial ones were examined for the photodegradation of cationic pollutants, pyridine chloride, and Rhodamine 6G. The mechanical stability of the titania powders prepared by EISA method were found to be superior to several commercial samples, however a NaOH post-treatment method was required. This, however, had a negative effect on the photocatalytic activity [154].

In general, the studies on mesoporous TiO_2_ materials prepared by EISA method point to the positive attributes of having large, uniform mesopores, and relatively high crystallinity in the TiO_2_ structure for photocatalytic degradation reactions.

### 4.3. Photocatalytic Splitting of Water

**Figure 25.**
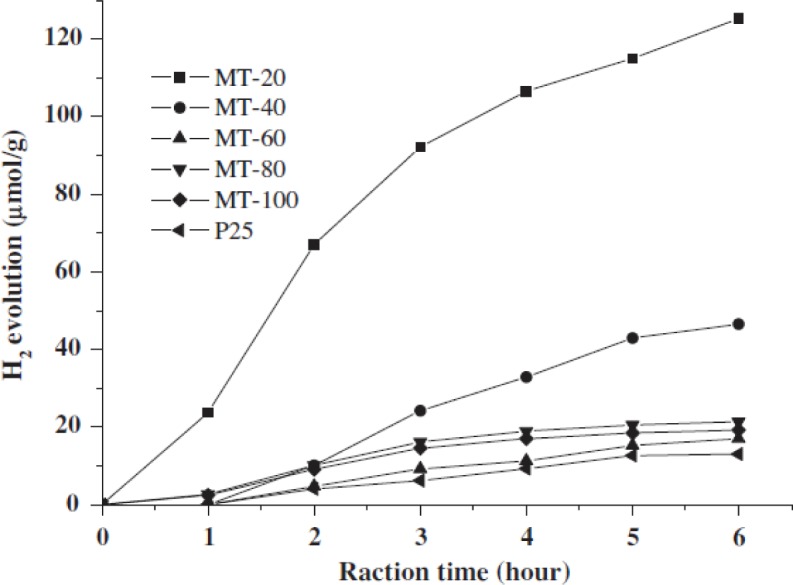
Depiction of solar-hydrogen production using various mesoporous TiO_2_ materials. Reprinted with permission from [53]. Copyright 2012 Elsevier.

The photocatalytic splitting of water to generate hydrogen as a fuel is non-polluting, environmentally friendly, and a carbon neutral method to alleviate the environmental impact of using fossil fuels [155,156]. Mesoporous TiO_2_ materials prepared by the EISA method have been used for photocatalytic splitting of water only in recent years and only a handful of reports are available. It should be pointed out that there are several reports of the application of mesoporous TiO_2_ prepared using surfactants, for photocatalytic splitting of water, but the vast majority of them were not prepared by the EISA method. The major challenge in using the EISA method seem to be in obtaining highly crystalline TiO_2_, while retaining the periodicity of the pores, since the crystallization of titania often collapses the pores. Liu and co-workers [53] produced mesoporous TiO_2_ nanoparticles by the EISA method and examined the solar hydrogen production using methanol-water mixture, and they obtained 120 μmol of H_2_ after 6 h of irradiation, as shown in Figure 25.

The material that had a pore diameter of 9.5 nm with an anatase crystallite size of 17.3 nm produced the highest H_2_ yield. The high activity of this material was attributed to the good crystallinity and the presence of an optimal size of titania crystallites at which surface and volume recombination of the charge-carriers are optimal [157]. Anatase TiO_2_ with smaller pore diameter and crystallite size yielded less hydrogen, perhaps due to enhanced surface recombination of electron-hole pairs. Park and co-workers [139] indicated that surface area plays a major role and observed hydrogen evolution rate of 160 μmol in 200 min. in their highest active mesoporous TiO_2_ powders. Wu and co-workers [126] applied EISA synthesis to produce both pillared and nanotube films of TiO_2_. However, the activity was modest with a production of approximately 40 μmol of H_2_ in 8 h. The photoelectrochemical properties of mesoporous sol-gel derived mesoporous TiO_2_ nanoparticle was found to be ten times higher than that of nanocrystalline TiO_2_ [158].

The application of mesoporous TiO_2_ materials for solar assisted splitting of water is relatively new. Increased textural properties such as high surface area and large pore diameter coupled with very good crystallinity of the TiO_2_ phase will favor solar hydrogen production using EISA prepared mesoporous TiO_2_ materials in the near future. Finally, it should be noted that the EISA approach is a simple and facile method that can be potentially extended to the preparation of other semiconductor photocatalysts such as WO_3_, Nb_2_O_5_, *etc.*

### 4.4. Batteries

**Figure 26.**
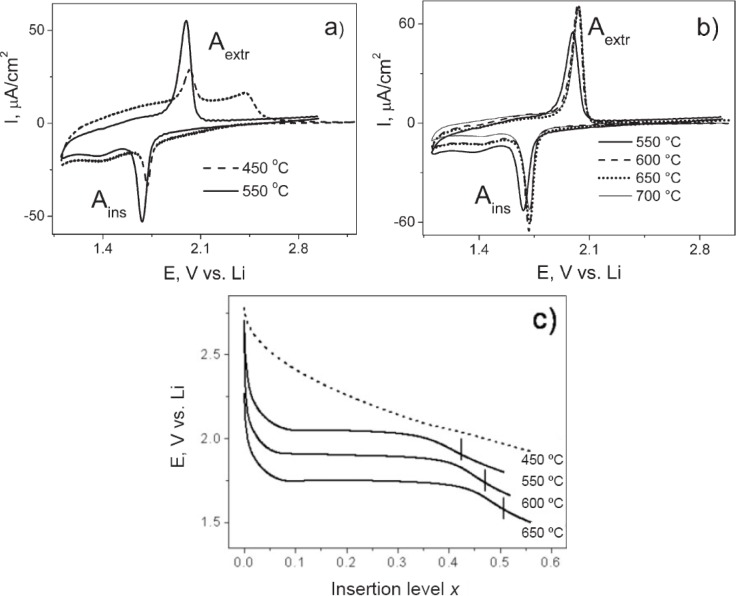
Cyclic voltammograms of mesoporous TiO_2_ materials prepared using KLE surfactant and calcined at (**a**) 450 and 550 °C; (**b**) in the temperature range of 550–700°C; and (**c**) galvanostatic insertion curves. Reprinted with permission from [54]. Copyright 2007 John Wiley and Sons.

The development of mesoporous TiO_2_ materials prepared by the EISA method for battery applications has occurred more recently. Electrochemical measurements, such as Cyclic Voltammograms (CVs), provide information on the electron transport properties similar to Electrochemical Impedance Spectroscopy (EIS). Electrochemical studies provide clues on the nature of the different phases and their periodicity [159,160]. Figure 26 shows the lithium insertion properties CVs for mesoporous TiO_2_ materials prepared by the EISA method [54]. Figure 26a shows the CVs at a scan rate of 0.5 mV/s for mesoporous TiO_2_ films made by the EISA method using non-ionic KLE surfactant. Two different calcination temperatures of 450 °C and 550 °C were used. The mesoporous TiO_2_ sample calcined at 450 °C has two Li extraction peaks (A_extr_) at 2.0 V and 2.4 V as shown in Figure 26a. This suggests that there are two phases (a predominant amorphous phase and an anatase phase) in the material giving rise to two different electron diffusion coefficients. In contrast, the sample calcined at 550 °C has a relatively single sharp Li extraction peak closer to 1.9 V. This indicates the presence of a highly crystalline material with one electron diffusion coefficient. The insertion and extraction of Li ions in TiO_2_ is a one-electron process, and ideally, there should be only one peak in the anodic and cathodic curves. The presence of two peaks in the sample calcined at 450 °C indicate the presence of an additional phase, *i.e.*, an amorphous one. Figure 26b shows the Li insertion and extraction peaks as a function of the calcination temperature in the range of 550–700 °C. As, the calcination temperature is increased, the thermodynamically stable rutile phase begins to form and this is indicated in the changes in the peak positions in the current-potential curves. The peak positions indicate the type of TiO_2_ phase.

Anatase TiO_2_ peak range is from 1.75 to 1.95 V with maximum at 1.85 V. Rutile TiO_2_ maximum is at 1.45 V, and TiO_2_(B) falls in the range of 1.5–1.6 V. Therefore, the peak height and position in the CVs can provide information regarding the crystallinity and the presence of TiO_2_ phase(s) that is confirmed directly by powder XRD studies. Figure 26c shows the galvanostatic insertion curves, which further indicate that after calcination at temperatures above 550 °C, the anatase structure is 100% crystalline. Bein and co-workers used P123 and *tert*-butanol in synthesis of mesoporous TiO_2_, by EISA method and a combination of anatase and TiO_2_(B) phases were formed [127]. The results from Bein and co-workers [127] further supports the effects of calcination temperature and type of non-ionic surfactant in producing mesoporous TiO_2_ materials with different phases. Finally, TiO_2_ could potentially be an inexpensive alternative material for battery storage applications.

## 5. Concluding Remarks

The EISA synthesis method is a simple and facile method to produce inorganic-organic hybrid mesoporous materials in the form of powders and thin films using various surfactants. The phase and crystallinity of titania formed is dependent on the nature of the surfactant and TiO_2_ precursor, the amount of water, pH, aging time, RH, and calcination temperature. A major challenge seems to be in obtaining mesoporous TiO_2_ that are highly uniform, having highly ordered pores, and simultaneously having high crystallinity. Calcination to remove the surfactants invariably leads collapse of pores and this may be prevented by use of pre-treatments by use of NH_4_OH and/or multi-step thermal treatments. The use of non-ionic surfactants seem to be beneficial in comparison to a cationic surfactant. This review will provide the reader with a rich history and perspective of the development of TiO_2_ mesoporous materials, and an insight into the EISA synthesis mechanism. Furthermore, the synthesis of highly ordered mesoporous TiO_2_ is greatly affected by small changes in the experimental condition(s) and this was discussed in great depth as well. Also, discussed were the variety of characterization techniques; this allows the reader to make a choice depending on the application, necessity, and availability, to completely characterize the resulting material and obtain a structure-activity relationship. The application of mesoporous TiO_2_ prepared by EISA method seem to be mostly devoted to photocatalytic degradation of organics and DSSC, its potential for solar assisted water splitting and battery storage applications have not been fully explored. It is with anticipation that this review was produced, hoping to spur new ideas and perhaps new directions and applications.
